# The *Shigella* Spp. Type III Effector Protein OspB Is a Cysteine Protease

**DOI:** 10.1128/mbio.01270-22

**Published:** 2022-05-31

**Authors:** Thomas E. Wood, Kathleen A. Westervelt, Jessica M. Yoon, Heather D. Eshleman, Roie Levy, Henry Burnes, Daniel J. Slade, Cammie F. Lesser, Marcia B. Goldberg

**Affiliations:** a Department of Medicine, Division of Infectious Diseases, Massachusetts General Hospital, Boston, Massachusetts, USA; b Department of Microbiology, Blavatnik Institute, Harvard Medical School, Boston, Massachusetts, USA; c Department of Immunology and Infectious Diseases, Harvard T. H. Chan School of Public Health, Boston, Massachusetts, USA; d Department of Molecular and Cellular Biology, Harvard University, Cambridge, Massachusetts, USA; e Department of Chemistry and Chemical Biology, Harvard University, Cambridge, Massachusetts, USA; f Department of Biochemistry, Virginia Polytechnic Institute and State University, Blacksburg, Virginia, USA; University of Massachusetts Amherst

**Keywords:** OspB, Shigella, TORC1, Tco89p, catalytic dyad, cysteine protease, inositol hexakisphosphate

## Abstract

The type III secretion system is required for virulence of many pathogenic bacteria. Bacterial effector proteins delivered into target host cells by this system modulate host signaling pathways and processes in a manner that promotes infection. Here, we define the activity of the effector protein OspB of the human pathogen *Shigella* spp., the etiological agent of shigellosis and bacillary dysentery. Using the yeast Saccharomyces cerevisiae as a model organism, we show that OspB sensitizes cells to inhibition of TORC1, the central regulator of growth and metabolism. *In silico* analyses reveal that OspB bears structural homology to bacterial cysteine proteases that target mammalian cell processes, and we define a conserved cysteine-histidine catalytic dyad required for OspB function. Using yeast genetic screens, we identify a crucial role for the arginine N-degron pathway in the yeast growth inhibition phenotype and show that inositol hexakisphosphate is an OspB cofactor. We find that a yeast substrate for OspB is the TORC1 component Tco89p, proteolytic cleavage of which generates a C-terminal fragment that is targeted for degradation *via* the arginine N-degron pathway; processing and degradation of Tco89p is required for the OspB phenotype. In all, we demonstrate that the *Shigella* T3SS effector OspB is a cysteine protease and decipher its interplay with eukaryotic cell processes.

## INTRODUCTION

Cellular processes are largely controlled by the availability of nutrients and the ability to respond to these environmental cues. Consequently, homeostatic control of metabolism is crucial to function, growth, and ultimately viability. The balance between anabolic and catabolic processes in eukaryotes is controlled by the target of rapamycin (TOR) complexes (TORC), two large multi-subunit hub-integrating sensory inputs to regulate cellular metabolism. In nutrient-replete conditions, amino acid and glucose availability sustains the kinase activity of the TORC1 complex, promoting translation, gene expression, and protein stability. In contrast, cellular stresses and starvation inhibit TORC1-dependent growth and stimulate proteolytic mechanisms such as autophagy to maintain amino acid pools (1).

Infection perturbs cellular homeostasis (2). Pathogens that invade host cells disrupt cellular processes in ways that promote the survival and replication of the infectious agent. *Shigella* spp. are the etiological agent of bacillary dysentery and a leading contributor to diarrheal mortality (3). This pathogen invades the intestinal epithelium, establishing a replicative niche within colonic epithelial cells and triggering an acute inflammatory immune response (4). The type III secretion system (T3SS) is required for S. flexneri infection, facilitating invasion and bacterial replication through the delivery into host cells of effector proteins that subvert cellular signaling pathways. Effector proteins also promote the spread of intracellular *Shigella* between cells, whereby it disseminates throughout the intestinal epithelium (5).

*Shigella* T3SS effector proteins display a myriad of well-characterized enzymatic activities, including phosphatase, acyltransferase, ubiquitin ligase and protease functions, as well as catalyzing other more unconventional biochemical modifications (6–12). The effector OspB has been described by our group and others as manipulating mTORC1 signaling, dampening the innate immune response *via* MAP kinase and NF-κB signaling, and modulating cytokine release (13–15). However, the precise mode of action of this effector protein is unknown.

In this study, we determine that the S. flexneri T3SS effector OspB is a cysteine protease. *In silico* analysis indicated structural homology to bacterial cysteine proteases, permitting identification of putative catalytic residues. Using a yeast model to study the impact of OspB activity on a eukaryotic host (16–20), we determine host factors required for OspB-dependent hypersensitivity to TORC1 inhibition, including inositol phosphate biosynthesis, TORC1 signaling, and protein degradation pathways. We find that the OspB-dependent hypersensitivity phenotype is due to the cleavage of the TORC1 component Tco89p, in a manner that requires both the conserved catalytic dyad in OspB and the secondary messenger molecule inositol hexakisphosphate. Finally, we demonstrate that the C-terminal product of Tco89p cleavage enters the arginine N-degron pathway for destruction by the proteasome, and that its degradation is required for OspB-dependent growth inhibition of yeast.

## RESULTS

### OspB exhibits structural homology to cysteine proteases.

**FIG 1:**
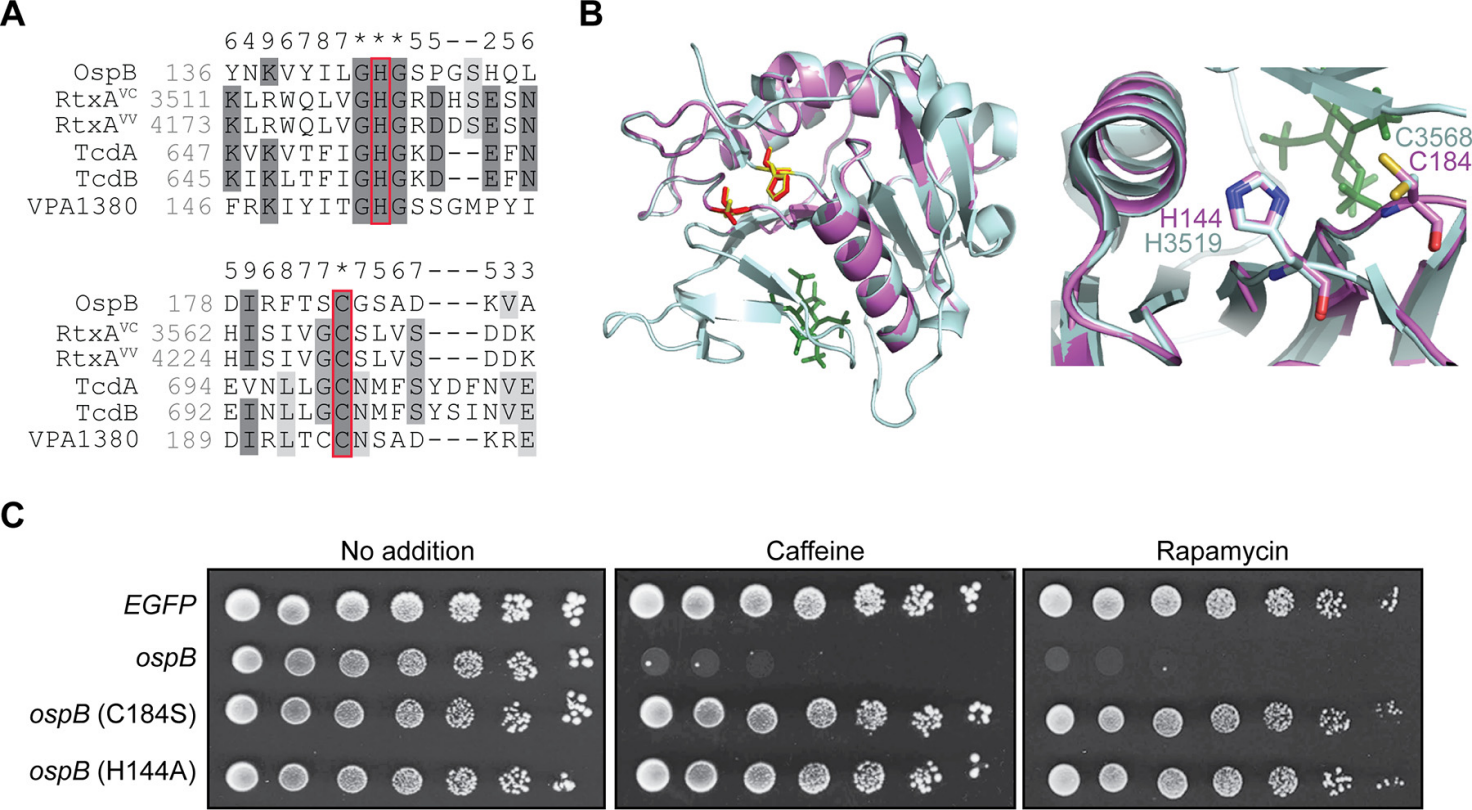
OspB possesses a predicted cysteine protease catalytic dyad. (A) Multiple sequence alignment of OspB with the catalytic sequences of the cysteine protease domains of RtxA from V. cholerae (RtxA^VC^) and V. vulnificus (RtxA^VV^), and autoprocessing domains of C. difficile TcdA and TcdB, and the OspB V. parahaemolyticus ortholog VPA1380. Red boxes indicate catalytic residues of the cysteine protease domains aligned with the putative catalytic residues of OspB. Darkness of gray shading reflects the conservation of individual residues, and the numbers above the alignment score the conservation at each position. Asterisks denote full conservation among the aligned sequences. (B) Cartoon depiction of a tertiary structure model of OspB (violet) on the structure of the cysteine protease domain of RtxA^VC^ (PDB: 3EEB)(pale cyan). Left panel: the catalytic residues of the RtxA^VC^ cysteine protease domain are denoted by yellow sticks and the putative catalytic residues of OspB shown as red sticks. In the RtxA^VC^ structure, the inositol hexakisphosphate cofactor is shown as green sticks. Right panel: an enlarged and rotated view shows the active site, highlighting the superposition of the putative OspB catalytic residues with those of the RtxA^VC^ cysteine protease domain, labeled according to the color of the cartoon. (C) Growth of yeast strains expressing wild-type *ospB*, and *ospB* constructs with mutation of a putative catalytic site residue, or the control *EGFP*. Serial dilutions spotted on media without additive or supplemented with a TORC1 inhibitor, caffeine or rapamycin (*n *= 3).

To gain insight into the potential mechanism of action of OspB, we performed Phyre2 analysis of the OspB sequence, which predicted structural similarity to the cysteine protease domains of the RtxA multifunctional autoprocessing repeats-in-toxin (MARTX) toxins of Vibrio cholerae and V. vulnificus. Further analysis revealed that OspB shares 27–30% sequence identity with the RtxA toxins and the protease domains of the large clostridial cytotoxins TcdA and TcdB of Clostridioides difficile (Fig. 1A). TcdA, TcdB, and RtxA are modular toxins that upon host cell endocytosis undergo autoproteolysis, which releases toxin domains that subvert cellular processes by inducing actin depolymerization and altering GTPase signaling (21, 22). In contrast to these large cytotoxins, OspB is small (288 amino acids; 32 kDa), and we found no evidence for OspB autoprocessing in cells (see Fig. S1 in the supplemental material).

10.1128/mbio.01270-22.1 FIG S1 Absence of evidence of processing of OspB in cell lysates. (A) Wild-type (WT) OspB and OspB (C184S) expressed in yeast, detected by immunoblotting with anti-OspB and anti-FLAG antibodies after separation on a 15% SDS-PAGE gel. α-tubulin serves as a loading control. (B) Samples from panel (A) separated on a 7.5% SDS-PAGE gel and probed as in panel (A). (C) Transfection of mouse embryonic fibroblasts with pCMV-*myc-ospB*. Cells were treated with rapamycin (10 nM) (rapa) or a DMSO vehicle control. Myc-OspB detected by immunoblotting with an anti-myc antibody after separation on a 10% SDS-PAGE gel. β-actin serves as the loading control; bands from a single blot. MW in kDa to the left of blots. Download FIG S1, PDF file, 0.6 MB. Copyright © 2022 Wood et al. 2022 Wood et al. https://creativecommons.org/licenses/by/4.0/ This content is distributed under the terms of the Creative Commons Attribution 4.0 International license.

The cysteine and histidine residues required for the proteolytic activity of the cysteine protease domains are conserved in OspB and the orthologous T3SS effector protein of V. parahaemolyticus VPA1380 (23–25) (Fig. 1A). Indeed, the tertiary structure of OspB can be modeled on the protease domains of RtxA and TcdA with 96% and 62% confidence, respectively, with conservation of the positions of their catalytic residues with C184 and H144 of OspB (Fig. 1B and Fig. S2A). The alignment of OspB with the cysteine protease domain structures suggested that OspB residues C184 and H144, may be required for OspB activity. A quantitative assay in yeast strains expressing S. flexneri effector proteins previously demonstrated that OspB causes growth inhibition of yeast in the presence of the cellular stressor caffeine (18). We utilized this assay to probe the role of these putative catalytic residues in OspB activity. Whereas expression of wild-type OspB elicited a drastic growth defect in the presence of caffeine, mutation of either C184 or H144 completely abrogated toxicity (Fig. 1C). These data indicate that OspB inhibition of yeast growth depends on the predicted catalytic dyad of C184 and H144, bolstering the prediction, based on the predicted tertiary structure of OspB, that it may be a structural homolog of the cysteine protease domains of several modular bacterial toxins.

10.1128/mbio.01270-22.2 FIG S2 The aspartic acid residues Asp108, Asp109, and Asp110 are not required for OspB activity. (A) Cartoon depiction of a tertiary structure model of OspB (violet) on the autoprocessing domain of TcdA (PDB: 3HO6) (pale cyan). Left panel: the catalytic residues of the cysteine protease domain are denoted by yellow sticks, with the putative catalytic residues of OspB shown as red sticks. The inositol hexakisphosphate cofactor in the TcdA protease domain structure is shown as green sticks. Right panel: an enlarged and rotated view shows the active site, highlighting the superposition of the putative OspB catalytic residues with those of the TcdA protease domain, labelled according to the color of the cartoon. (B) Multiple sequence alignment of OspB with the aspartic acid residue of the cysteine protease domains of RtxA from V. cholerae (RtxA^VC^) and V. vulnificus (RtxA^VV^) and C. difficile TcdA and TcdB, which in some cases stabilizes the catalytic histidine residue (1, 2). Also shown is the OspB ortholog V. parahaemolyticus VPA1380. The red box indicates the aspartic acid residue in the CPDs. Darkness of gray shading reflects the conservation of individual residues, and the numbers above the alignment score the conservation at each position. (C) Growth of yeast strains expressing *ospB* constructs or an empty vector control. Serial dilutions spotted on media without additive or supplemented with a TORC1 inhibitor, caffeine or rapamycin (*n *= 3). Download FIG S2, TIF file, 13.2 MB. Copyright © 2022 Wood et al. 2022 Wood et al. https://creativecommons.org/licenses/by/4.0/ This content is distributed under the terms of the Creative Commons Attribution 4.0 International license.

Mutagenesis studies of TcdA showed that in addition to C700 and H655, D589 is required for autoprocessing through proton abstraction from the histidine in the active site (26). In RtxA, mutagenesis of the equivalent aspartic acid residue D3469 alone does not impact proteolytic activity; however, substitution of E3467 and D3469 together results in partial loss of autocleavage (27). In OspB, an aspartic acid residue (D108) is predicted to be present at the equivalent position of D589^TcdA^, adjacent to two additional aspartic acid residues, D109 and D110 (Fig. S2A and S2B). These were therefore candidates for involvement in catalysis. Alanine substitution of none of these aspartic acid residues individually rescued yeast growth (Fig. S2C). In addition, a D108A/D110A double mutant had no effect on OspB activity. These results indicate that the function of OspB requires cysteine and histidine residues, analogous to the cysteine-histidine catalytic dyad of the cysteine protease domain of RtxA.

Among the effects of caffeine on cellular processes, inhibition of TORC1 is described as an important mode of action for it in yeast (28). To determine whether the effect of caffeine on the OspB phenotype is specific to TORC1, we replaced caffeine in the media with rapamycin, which unlike caffeine is a specific inhibitor of TORC1. As with caffeine, the presence of rapamycin sensitized yeast to growth inhibition by OspB in a manner that depended on residues C184 and H144 (Fig. 1C). These data indicate that the impact of OspB on yeast growth depends on inhibition of TORC1, consistent with our previous findings that OspB potentiates rapamycin inhibition of growth of mammalian cells (29). The presence of a similar OspB-dependent phenotype in yeast and mammalian cells with respect to sensitization to TOR inhibition demonstrates that yeast present a reasonable model for investigating the mechanism of OspB activity.

### Inositol hexakisphosphate is required for OspB activity.

**FIG 2:**
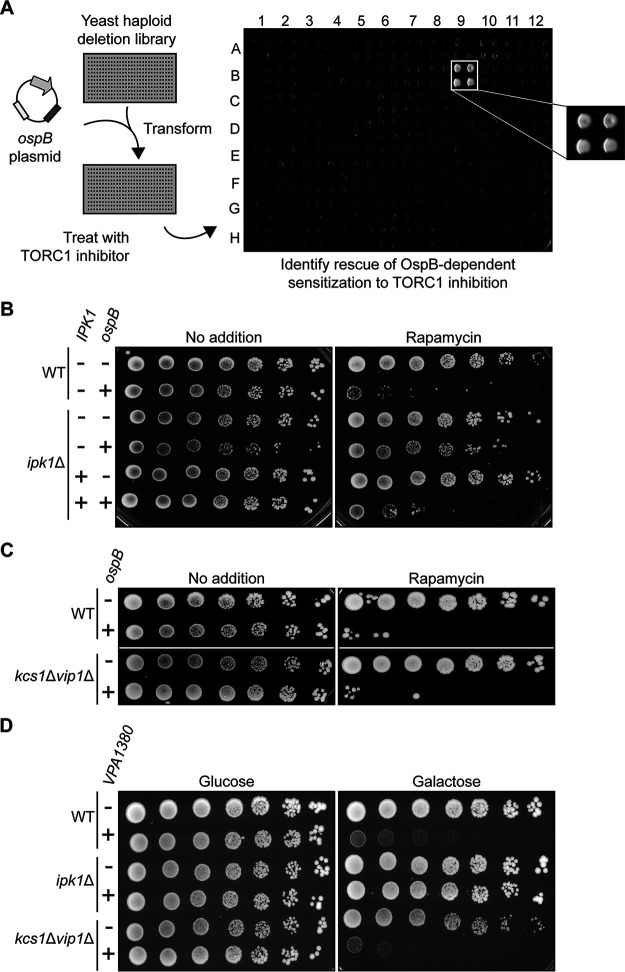
Inhibition of yeast growth by OspB-family effectors requires inositol hexakisphosphate. (A) Schematic of the deletion library screen designed to identify host factors in yeast required for OspB-mediated growth inhibition in the presence of caffeine. An example of an output plate is shown, spotted in quadruplicate, with one hit (magnified inset). (B) Growth of wild-type (WT) or *ipk1* deletion yeast strain, each expressing *ospB* constructs or vector control. *IPK1* encodes inositol 1,3,4,5,6-pentakisphosphate 2-kinase, the enzyme responsible for IP_6_ production. Serial dilutions spotted on media with or without rapamycin. (C) Growth of WT or *kcs1 vip1* yeast strain, each expressing *ospB* constructs or vector control. *KCS1* and *VIP1* encode the two inositol hexakisphosphate kinases. Serial dilutions spotted on media with or without rapamycin. (D) Growth of yeast strains described in (B) and (C), each expressing *VPA1380* or vector control. Serial dilutions spotted on media, in conditions that repress (glucose) or induce (galactose) *VPA1380* expression.

With the goal of identifying host factors required for OspB activity, we screened a S. cerevisiae deletion library for strains in which OspB was no longer able to inhibit yeast growth in the presence of caffeine (Fig. 2A). The OspB-mediated growth defect was diminished in the absence of 81 genes, including several whose gene products act downstream of TORC1 (Table S1). Deletion of these genes would be expected to uncouple TORC1 signaling from its downstream transcriptional response, thereby decreasing the sensitivity to TORC1 inhibitors. This finding validates the ability of the screen to identify host factors required for the OspB-mediated growth phenotype and confirms a role of TORC1 signaling therein.

10.1128/mbio.01270-22.8 TABLE S1 Genes identified as required for OspB-dependent sensitization of yeast to caffeine. Individual yeast strains lacking each of these genes displayed robust growth when expressing *ospB* in the presence of caffeine. Robust growth was defined quantitatively as previously described (3). Genes encoding proteins with a characterized role in TORC1 signaling are shown in bold. Download Table S1, PDF file, 0.1 MB. Copyright © 2022 Wood et al. 2022 Wood et al. https://creativecommons.org/licenses/by/4.0/ This content is distributed under the terms of the Creative Commons Attribution 4.0 International license.

The screen also identified *IPK1*, which encodes the enzyme responsible for the generation of inositol hexakisphosphate (IP_6_), as required for yeast growth inhibition by OspB. Using an independent *ipk1* deletion strain, we confirmed that *IPK1* is required for OspB-mediated growth sensitivity to TORC1 inhibitors and found that reintroduction of *IPK1* in *trans* restored growth inhibition (Fig. 2B). Of note, IP_6_ is an activator of the protease domains of RtxA and TcdA, and it is required for their cysteine protease activity *in vitro* (27, 30–33). *In vitro* data assessing the role of IP_6_ and a more highly phosphorylated inositol pyrophosphate species (IP_7_) in the autoprocessing of TcdB indicate that IP_7_ is also a potent activator of the TcdB protease activity (34). Since an *ipk1* mutant lacks IP_6_ and all IP_7_ and IP_8_ inositol pyrophosphate species (35), we tested whether these inositol pyrophosphatase species were dispensable for OspB enzymatic activity by assessing growth inhibition in the absence of both yeast inositol hexakisphosphate kinases Kcs1p and Vip1p (36). The *kcs1*Δ*vip1*Δ mutant still displayed OspB-dependent sensitivity to TORC1 inhibitors, indicating that IP_6_ is sufficient to stimulate the activity of OspB (Fig. 2C). Furthermore, we confirmed previous data (23) that showed that IP_6_ is sufficient for enzymatic activation of the OspB ortholog VPA1380 from Vibrio parahaemolyticus (Fig. 2D), pointing to the Ipk1p-dependency of VPA1380 for yeast growth inhibition resulting specifically from the loss of IP_6_ rather than inositol pyrophosphate species.

### The arginine N-degron pathway is required for growth inhibition by OspB.

**FIG 3:**
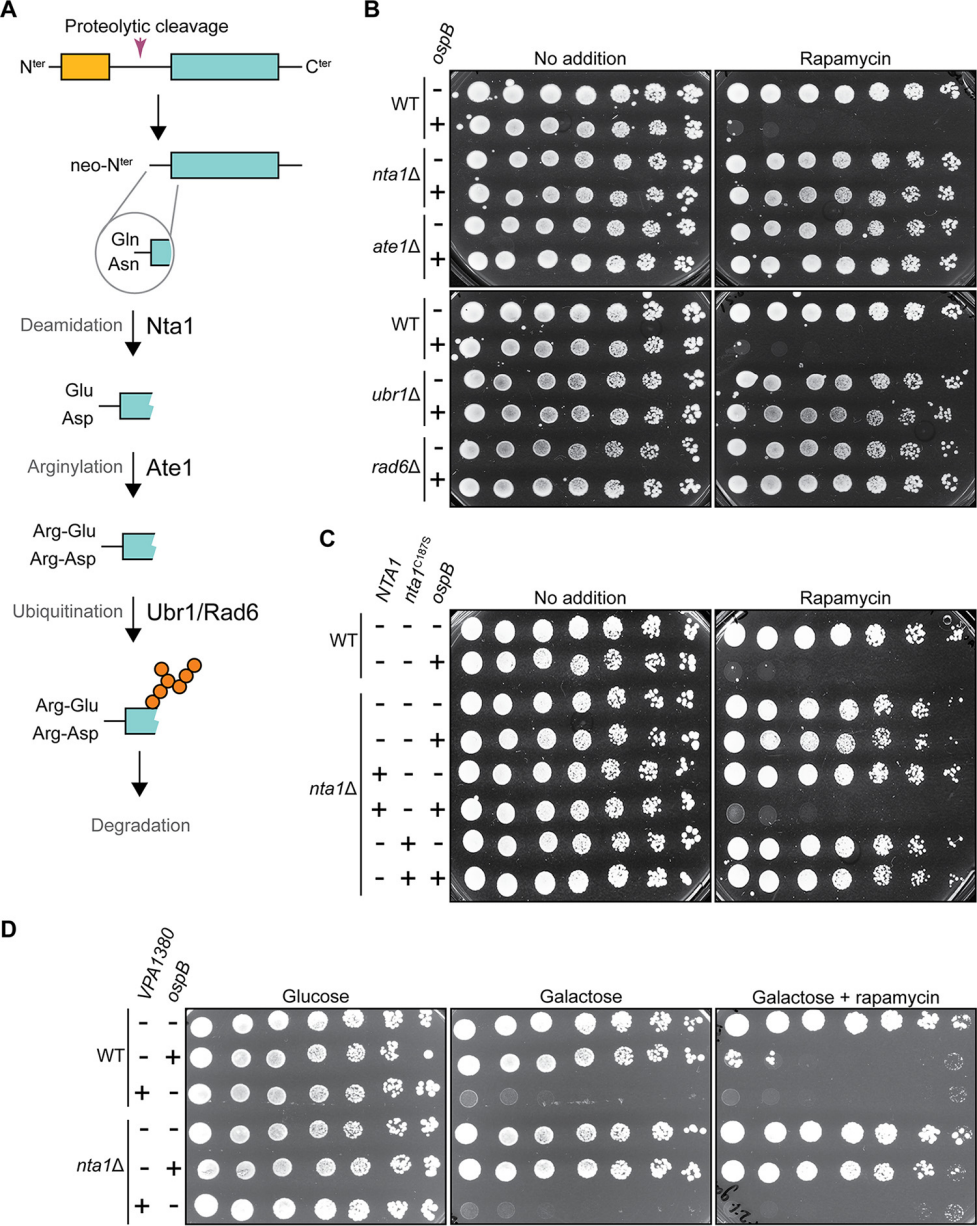
The arginine N-degron pathway is required for growth inhibition by OspB. (A) Schematic of the arginine N-degron pathway. (B) Growth of wild-type (WT) yeast or yeast strains containing deletions of genes encoding components of the arginine N-degron pathway, each expressing *ospB* or vector control. Serial dilutions were spotted on media with or without rapamycin (*n *= 3). (C) Growth of WT or *nta1* deletion yeast strain, each expressing *ospB* or vector control, complemented or not with a functional (*NTA1*) or catalytically inactive (*nta1*^C187S^) *NTA1* allele. Serial dilutions spotted on media with or without rapamycin (*n *= 3). (D) Growth of WT or *nta1* deletion yeast strain expressing *ospB*, *VPA1380*, or vector control. Serial dilutions spotted on media with or without rapamycin, in conditions that repress (glucose) or induce (galactose) *VPA1380* expression (*n *= 3).

Our deletion screen for host factors required for OspB-mediated sensitization of yeast to TORC1 inhibition also identified several components of the arginine N-degron pathway. N-degron pathways recognize the neo-N termini of polypeptides generated by cleavage or processing events and targets the polypeptides to the proteasome (37). If the N-terminal residue of the C-terminal fragment produced upon protein cleavage is Gln or Asn, this destabilizes the fragment, directing its degradation *via* the arginine N-degron pathway (38). The N-terminal Gln or Asn residue is deamidated to a Glu or Asp residue, respectively, by the N-terminal amidase Nta1p. The polypeptide is arginylated at the N terminus by the arginine transferase Ate1p, permitting subsequent recruitment of the E3-E2 ubiquitin ligase N-recognin complex Ubr1p-Rad6p, which ubiquitinates the N-degron for degradation by the proteasome (Fig. 3A) (39–42).

Deletion of *nta1*, *ate1*, *ubr1*, or *rad6* each rescued the growth inhibition phenotype catalyzed by OspB (Fig. 3B). Moreover, complementation with *NTA1* rescued OspB-dependent sensitivity to TORC1 inhibition in the *nta1* mutant, whereas complementation with an *nta1* (C187S) allele, encoding a catalytically inactive amidase, did not (Fig. 3C and Fig. S3). These results show that the arginine N-degron pathway is required for growth inhibition by OspB and suggests that the generation of an N-degron harboring an N-terminal Gln or Asn is a necessary step in this process.

10.1128/mbio.01270-22.3 FIG S3 Production of Nta1p variants. Abundance of the WT and catalytically inactive Nta1p variant in cells that express OspB or GFP control. Western blot. Asterisk, a nonspecific protein recognized by the anti-OspB antibody. α-tubulin, loading control (*n *= 3). Download FIG S3, PDF file, 0.4 MB. Copyright © 2022 Wood et al. 2022 Wood et al. https://creativecommons.org/licenses/by/4.0/ This content is distributed under the terms of the Creative Commons Attribution 4.0 International license.

In a parallel screen using a yeast overexpression library (43) to identify suppressors of OspB-mediated sensitivity to caffeine, we found that induction of expression of *BRE1* from a multicopy vector rescued the OspB-dependent growth defect (Table S2). Bre1p is an E3 ubiquitin ligase which, in conjunction with the E2 ubiquitin conjugating enzyme Rad6p, monoubiquitinates histone H2B to regulate chromatin structure (44, 45). We hypothesized that the mechanism of rescue of the OspB phenotype by Bre1 overexpression might be its sequestration of Rad6p, thereby decreasing the availability of Rad6p to the arginine N-degron pathway, effectively phenocopying a *rad6* mutant (Fig. S4A). We found that the E2 enzymatic activity of Rad6p is required for growth inhibition by OspB, since production of a catalytically inactive Rad6p (C88S) variant did not rescue the OspB phenotype in a *rad6* mutant, whereas the wild-type *RAD6* allele did (Fig. S4B). In contrast, overexpression of a catalytically dead Bre1p (C663S) variant rescued the OspB growth defect (Fig. S4C), indicating that the E3 ubiquitin ligase activity of Bre1p is dispensable for suppression of the OspB phenotype, consistent with the proposed mechanism of suppression being Rad6p sequestration. Expression of an additional copy of *RAD6* negated the suppression phenotype of Bre1p overexpression (Fig. S4D), suggesting that higher levels of Bre1p rescue growth through indirectly reducing flux through the arginine N-degron pathway. Taken together, these data demonstrate that the activity of the arginine N-degron pathway is critical for OspB to sensitize yeast to TORC1 inhibition.

10.1128/mbio.01270-22.4 FIG S4 Bre1p rescues growth inhibition through sequestration of arginine N-degron pathway component Rad6p. (A) Schematic of the proposed mechanism of Bre1-mediated suppression of OspB-dependent growth inhibition. (B) Growth of wild-type (WT) or *rad6* deletion yeast strains expressing *ospB* or vector control. Serial dilutions spotted on media with or without caffeine (*n *= 3). (C) Growth of yeast strains expressing *ospB* or vector control in the presence or absence of indicated multicopy *BRE1* alleles. Serial dilutions spotted on media with or without caffeine (*n *= 3). (D) Growth of WT yeast expressing *ospB* or vector control in the presence or absence of additional gene copies of *BRE1* and/or *RAD6*. Serial dilutions spotted on media with or without caffeine (*n *= 3). Download FIG S4, PDF file, 0.9 MB. Copyright © 2022 Wood et al. 2022 Wood et al. https://creativecommons.org/licenses/by/4.0/ This content is distributed under the terms of the Creative Commons Attribution 4.0 International license.

10.1128/mbio.01270-22.9 TABLE S2 Genes identified as suppressors of OspB-dependent sensitization of yeast to caffeine. Expression of these genes from a multicopy vector under an inducible promoter rescued the growth of wild-type yeast co-expressing *ospB* when grown in the presence of caffeine. Assessment of growth was conducted in a qualitative manner. Download Table S2, PDF file, 0.1 MB. Copyright © 2022 Wood et al. 2022 Wood et al. https://creativecommons.org/licenses/by/4.0/ This content is distributed under the terms of the Creative Commons Attribution 4.0 International license.

VPA1380 expression is toxic to yeast even in the absence of TORC1 inhibitors, indicative of a mechanism divergent to that of OspB. In addition, the absence of *nta1* or other arginine N-degron pathway components did not perturb the growth inhibition elicited by VPA1380, indicating that the outcome of its activity differs from that of OspB (Fig. 3D and Fig. S5). Furthermore, no role was found for the formyl-methionine or proline N-degron pathways (46–48) in VPA1380-mediated growth inhibition (Fig. S5). Thus, despite homology between OspB and VPA1380, and that both inhibit yeast growth, these data suggest that OspB and VPA1380 elicit toxicity *via* divergent mechanisms.

10.1128/mbio.01270-22.5 FIG S5 N-degron pathways are not required for VPA1380 toxicity phenotype, and VPA1380 does not cleave Tco89p. (A) The effect on growth of *ospB* and *VPA1380* expression in wild-type (WT) yeast, proline N-degron pathway mutants (lacking N-recognins Gid4 or Gid10), arginine N-degron pathway mutants (lacking amidase Nta1, E3 ubiquitin ligase Ubr1, or E2 conjugating enzymes Ubc5 or Rad6), or a formyl-methionine N-degron pathway mutant (lacking E3 ubiquitin ligase Psh1). Serial dilutions spotted on media in conditions that repress (glucose) or induce (galactose) construct expression. Supplementation of media with rapamycin where indicated (*n *= 2). (B) Absence of cleavage of Tco89p by VPA1380. Western blot. α-tubulin is the loading control (*n *= 2). Download FIG S5, PDF file, 1.2 MB. Copyright © 2022 Wood et al. 2022 Wood et al. https://creativecommons.org/licenses/by/4.0/ This content is distributed under the terms of the Creative Commons Attribution 4.0 International license.

### OspB cleaves the TORC1 component Tco89p.

**FIG 4:**
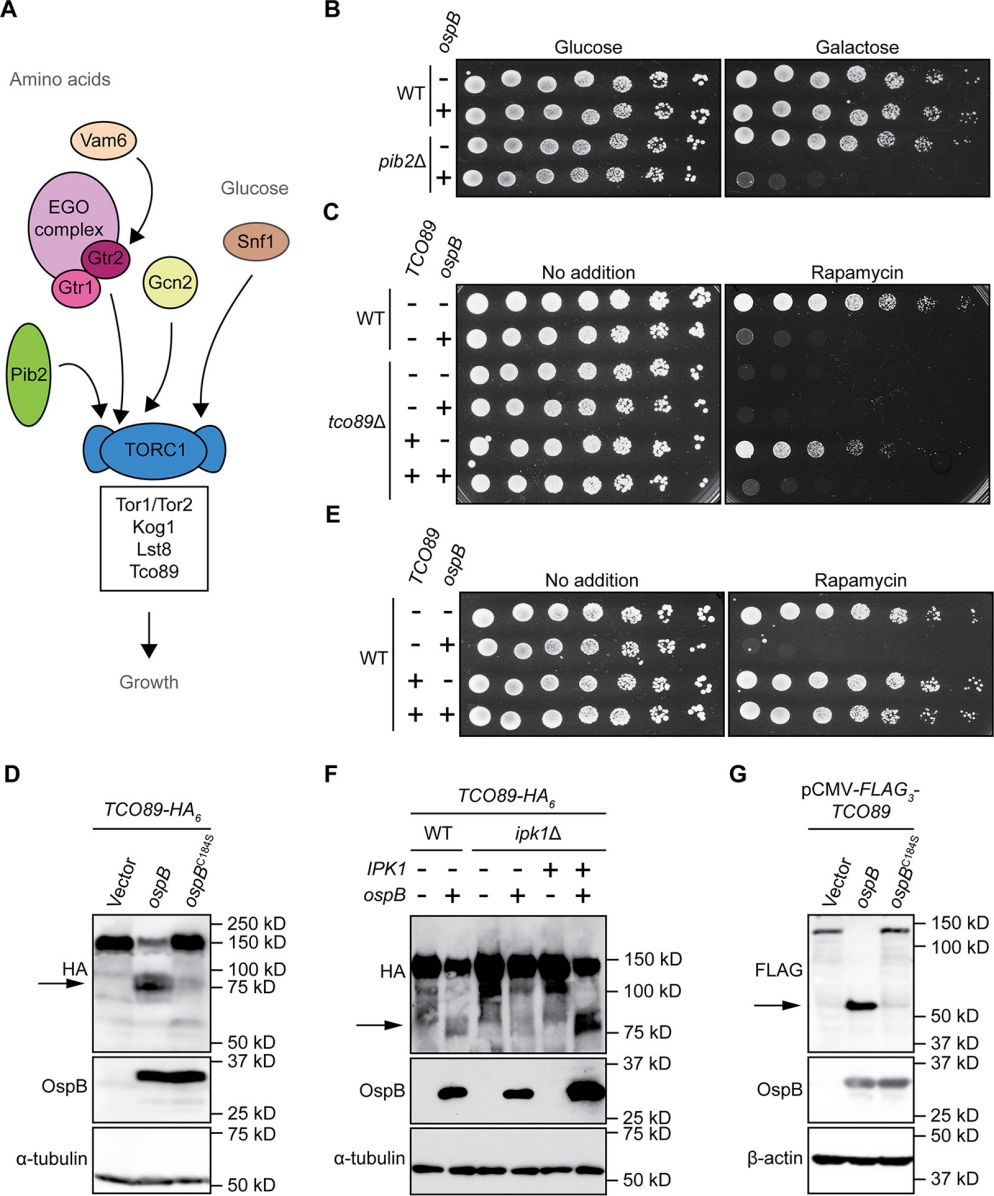
The TORC1 subunit Tco89p is cleaved by OspB. (A) Diagram of the yeast TORC1 signaling network. (B) Growth of wild-type (WT) or *pib2* deletion yeast strain, each expressing *ospB* or vector control. Serial dilutions spotted on media, in conditions that repress (glucose) or induce (galactose) *ospB* expression (*n *= 3). (C) Growth of WT or *tco89* deletion yeast strain, each expressing *ospB* or vector control. Serial dilutions spotted on media with or without rapamycin (*n *= 4). (D) Cleavage of Tco89p in yeast, in the presence of OspB, OspB(C184S), or vector control. Western blot. α-tubulin, loading control. Arrow, Tco89p C-terminal cleavage product (*n *= 4). (E) Growth of wild-type yeast expressing *ospB* or vector control, in the presence or absence of *TCO89* expression from a multicopy plasmid. Serial dilutions spotted on media with or without rapamycin (*n *= 3). (F) Cleavage of Tco89p by OspB in WT or *ipk1* deletion yeast strains. Western blot. α-tubulin, loading control. Arrow, Tco89p C-terminal cleavage product (*n *= 3). (G) Cleavage of a Tco89p construct co-expressed in HEK293T cells with OspB, OspB(C184S), or vector control. Western blot. β-actin, loading control. Arrow, Tco89p N-terminal cleavage product (*n *= 3).

Since yeast expressing OspB are sensitive to TORC1 inhibitors, we postulated that OspB either perturbs TORC1 signaling upstream of TORC1 or directly manipulates the TORC1 complex itself. Genetic ablation of each of the individual nutrient sensing pathways upstream of TORC1 (Fig. 4A) (49–53) did not rescue the OspB phenotype (Fig. S6A). With respect to *PIB2*, which encodes a glutamine sensor that activates TORC1 in parallel to the amino acid–responsive Gtr1p/Gtr2p pathway (54, 55), a yeast strain constitutively producing OspB in the absence of *PIB2* could not be generated; however, expression of *ospB* from a galactose inducible promoter completely inhibited the growth of a *pib2* mutant without the need for low levels of caffeine or rapamycin (Fig. 4B). Genetic ablation of components of TORC1 or the Gtr1p/Gtr2p branch of amino acid sensing is synthetically lethal in a *pib2*Δ background (53), and we found that OspB is still toxic in a *gtr1*Δ*gtr2*Δ double mutant (Fig. S6B). We therefore concluded that OspB likely targets the TORC1 complex itself.

10.1128/mbio.01270-22.6 FIG S6 OspB does not act upstream of TORC1. (A) Effect of *ospB* expression on the growth of yeast strains lacking genes involved in nutrient sensing upstream of TORC1 signaling compared with wild-type (WT) yeast. Serial dilutions spotted on media with or without rapamycin (*n *= 3). (B) Growth of WT and a *gtr*1Δ*gtr*2Δ yeast strain expressing *ospB*, plated on solid media containing no additive or 1.5 nM rapamycin. This reduced rapamycin concentration was used due to sensitivity of the *gtr*1Δ*gtr*2Δ mutant to it (*n *= 3). Download FIG S6, PDF file, 1.5 MB. Copyright © 2022 Wood et al. 2022 Wood et al. https://creativecommons.org/licenses/by/4.0/ This content is distributed under the terms of the Creative Commons Attribution 4.0 International license.

We tested whether any of the four proteins that comprise the TORC1 complex—Kog1p, Lst8p, Tco89p and Tor1p/Tor2p (56)—are perturbed by OspB activity. Among these four proteins, only Tor1p and Tco89p are nonessential. We found that neither the essential TORC1 components nor Tor1p is cleaved by OspB (Fig. S7A). Since deletion of *TCO89* rendered yeast hypersensitive to rapamycin (56), it was not possible to test for an effect of OspB using this growth assay. *TCO89* complementation restored TORC1 inhibitor sensitivity to wild-type levels (Fig. 4C).

10.1128/mbio.01270-22.7 FIG S7 Independence of OspB protease activity from components of TORC1 other than Tco89p and inositol pyrophosphates. (A) Lack of cleavage of components of the yeast TORC1 complex by OspB. TORC1 proteins (Tor1p, Tor2p, Kog1p and Lst8), tagged and at their native loci. The OspB construct produced by the *KOG1-HA_6_ LST8-MYC_9_* strain is untagged, whereas in all other strains, OspB has a C-terminal FLAG_3_-His_6_ tag. Western blot. α-tubulin, loading control (*n* =3). (B) Cleavage of Tco89p in wild-type and *kcs*1Δ*vip1*Δ yeast, in the presence of OspB, OspB(C184S) or vector control. Western blot. α-tubulin, loading control. Arrow, the Tco89p C-terminal cleavage product (*n *= 3). Download FIG S7, PDF file, 0.5 MB. Copyright © 2022 Wood et al. 2022 Wood et al. https://creativecommons.org/licenses/by/4.0/ This content is distributed under the terms of the Creative Commons Attribution 4.0 International license.

Assessment of Tco89p abundance revealed that in the presence of OspB, full-length Tco89p levels were substantially decreased and a faster migrating Tco89p band that was recognized by an antibody to the C-terminal tag was present (Fig. 4D). This faster migrating band was not observed in the presence of the catalytically inactive OspB C184S mutant, indicating that its generation depends on OspB catalytic activity and that the faster migrating Tco89p band is a C-terminal cleavage product. Overexpression of *TCO89* in wild-type yeast rescued the OspB-dependent growth defect (Fig. 4E), presumably because the resulting increase in Tco89p levels raised the threshold at which OspB-dependent sensitivity to TORC1 inhibitors occurs.

Cleavage of endogenous Tco89p was abolished in an *ipk1*Δ background and restored by complementation of *IPK1*, indicating that requirements for the OspB-mediated growth phenotype are associated with the Tco89p cleavage phenotype (Fig. 4F). Tco89p cleavage by OspB was unaffected in a *kcs1*Δ*vip1*Δ mutant (Fig. S7B), providing additional evidence that IP_6_ is the inositol phosphate species acting as the cofactor for OspB protease activity. Upon transfection of mammalian cells with both OspB and Tco89p, OspB cleavage of Tco89p occurred (Fig. 4G), which indicates that OspB functions as a protease in mammalian cells and suggests that Tco89p may be a direct substrate of OspB protease activity. Processing of Tco89p by VPA1380 was not observed (Fig. S5B), further supporting that the mechanisms of OspB and VPA1380 are divergent. Together, these data demonstrate that, in the presence of IP_6_, OspB cleaves the nonessential TORC1 component Tco89p, triggering sensitivity to inhibition of TORC1 signaling.

**FIG 5:**
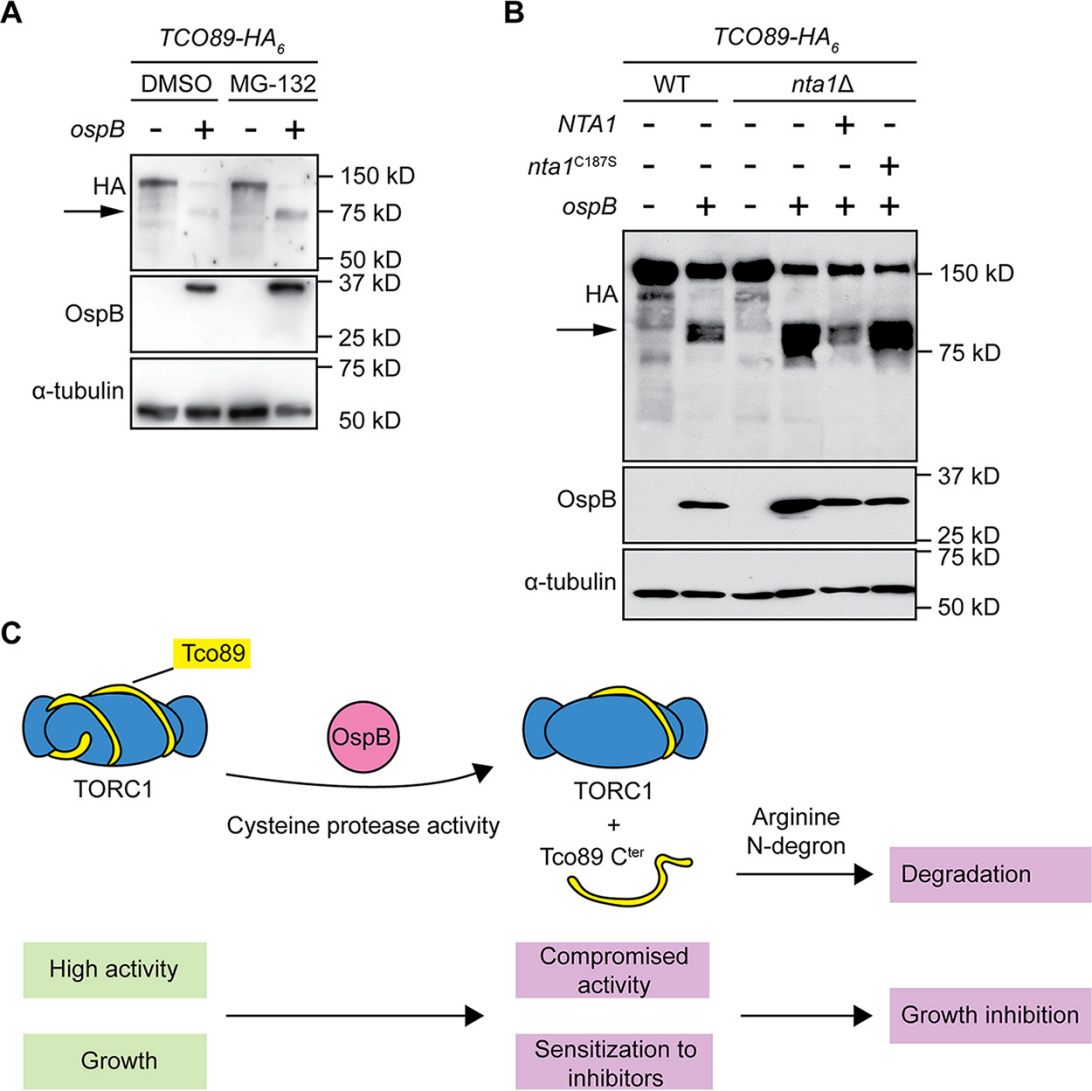
Degradation of the Tco89p C-terminal fragment by the arginine N-degron pathway. (A) Increase in abundance of Tco89p C-terminal cleavage product upon OspB cleavage in the presence proteasome inhibitor MG-132. Western blot. α-tubulin, loading control. Arrow, Tco89p C-terminal cleavage product (*n *= 3). (B) Increase in abundance of Tco89p C-terminal cleavage product upon OspB cleavage in the absence of a functional *NTA1* allele. α-tubulin, loading control. Arrow, Tco89p C-terminal cleavage product (*n *= 3). (C) Model of the mechanism of OspB-mediated sensitization of yeast to TORC1 inhibition. Blue shapes, TORC1; yellow spiral, Tco89p; pink circle, OspB.

We assessed the role of the arginine N-degron pathway in the stability of the C-terminal Tco89p cleavage product generated by OspB. Treatment of yeast with the proteasome inhibitor MG-132 increased the abundance of the Tco89p C-terminal fragment, indicating that this fragment is a substrate of the proteasome (Fig. 5A). Deletion of *NTA1* resulted in an increase in the abundance of the Tco89p C-terminal fragment (Fig. 5B), and its levels were restored by reintroduction of *NTA1*, but not by reintroduction of the *nta1* (C187S) inactive mutant, indicating that the C-terminal fragment of Tco89p is the N-degron that mediates growth inhibition caused by the protease activity of OspB.

In summary, we found that the *Shigella* T3SS effector OspB is a cysteine protease that cleaves the TORC1 component Tco89p, thereby generating an N-degron, and that the N-degron is targeted for degradation by the arginine N-degron pathway (Fig. 5C). Cleavage of Tco89p by OspB and host-mediated degradation of the C-terminal fragment is responsible for sensitization of the TORC1 complex to inhibition and the associated inhibition of yeast growth.

## DISCUSSION

The evidence presented here collectively demonstrates that the *Shigella* T3SS effector OspB is a cysteine protease and that it requires inositol hexakisphosphate for its activity. OspB is required for cleavage of Tco89p, a component of yeast TORC1, and upon expression of the two proteins in mammalian cells, OspB is sufficient to cleave Tco89p (Fig. 4G), indicating that OspB-mediated cleavage of Tco89p is either direct or depends on factors that are conserved between yeast and mammalian cells. OspB is structurally homologous to the cysteine protease domains of the bacterial cytotoxin RtxA, with conservation of the catalytic cysteine and histidine residues (Fig. 1 and Fig. S2) (24, 30, 31, 57). In OspB, the conserved cysteine and histidine are each required for both the OspB growth inhibition phenotype in yeast and Tco89p cleavage in yeast and mammalian cells (Fig. 4 and data not shown).

Like TcdA, TcdB and RtxA, OspB requires inositol hexakisphosphate for its activity (Fig. 2 and 4) (58). We postulate that OspB likely binds IP_6_, since although the tertiary structure of the lysine-rich IP_6_ binding pocket is not modeled by Phyre2 analysis (Fig. 1), OspB is a lysine- and arginine-rich protein (31 Lys and 8 Arg among 288 total residues). Furthermore, many of these positively charged residues are conserved in VPA1380 (data not shown) and are required for the yeast growth inhibition phenotype elicited by VPA1380 (23). Using a genetic approach, we exclude inositol pyrophosphate species as being necessary for OspB activity and define the inositol phosphate species requirement as IP_6_; however, it is possible that an inositol pyrophosphate species may be sufficient for OspB activity, as found for IP_7_ and C. difficile TcdB *in vitro* (34). The requirement for a host-specific cofactor, such as IP_6_, ubiquitin, calmodulin, or cyclophilins, for the activation bacterial effectors is increasingly appreciated (59–64). The evolutionary benefit of this is clear, as it necessarily restricts enzymatic activity to the context of host infection. Together, these findings provide strong evidence that OspB is a cysteine protease in the family of proteases represented by the cysteine protease domains of the MARTX toxins and large clostridial cytotoxins.

Two other *Shigella* T3SS effectors, IpaJ and OspD3, are also cysteine proteases. IpaJ and OspD3 are divergent from OspB, and their substrates are distinct—Rho GTPases and necroptotic signaling factors, respectively (65, 66). Genes encoding T3SS effector proteins are often acquired by horizontal gene transfer (67), thus homologous effectors are commonly secreted by the T3SSs of pathogens displaying similar host tropism. The T3SS2 effector of V. parahaemolyticus VPA1380 is homologous to OspB, yet we find that it likely targets a different host substrate. First, yeast growth inhibition by OspB requires reduction of TORC1 activity by either chemical or genetic intervention (Fig. 1 and 4), whereas VPA1380 is toxic to yeast in the absence of additional stressors (Fig. 2 and [23]). Second, we find that VPA1380 neither cleaves Tco89p nor generates a substrate cleavage product that requires the arginine N-degron pathway for its degradation (Fig. 3 and Fig. S5).

OspB cleavage of Tco89p, a component of TORC1, produces a C-terminal fragment that enters the arginine N-degron pathway for proteasomal degradation, rendering the cells hypersensitive to TOR inhibition. Tco89p cleavage and degradation appears to be entirely responsible for the TOR inhibitor hypersensitivity phenotype mediated by OspB, as degradation of the C-terminal fragment phenocopies a *tco89*Δ mutant growth defect in the presence of either rapamycin or caffeine (Fig. 4C). Consistent with this, complementation with multicopy Tco89p is associated with reduced sensitivity to inhibition of TORC1 by OspB in the presence of a chemical inhibitor (Fig. 4E). The dependence on *NTA1*, the upstream-most enzyme in the arginine N-degron pathway (Fig. 3), indicates that OspB cleavage results in a tertiary arginine N-degron, with Gln or Asn at the neo-N terminus of the C-terminal Tco89p cleavage product, since deamidation of the product by Nta1p is a critical step in its degradation (38).

The migration of Tco89p constructs in SDS-PAGE is slower (at around 150 kDa) than expected for the 89 kDa protein (Fig. 4). We hypothesize that the retarded migration of Tco89p is due to significant phosphorylation by the TORC1 kinase (68, 69). Irrespective of the cause, prediction of the OspB cleavage site producing the C-terminal fragment cannot be based on gel migration. Tco89p is an intrinsically disordered protein, and disordered proteins are often enriched in phosphorylation sites (70). Moreover, posttranslational modification is a frequent regulator of intrinsically disordered proteins, so it is conceivable that TORC1 regulates its own function by altering the phosphorylation state of Tco89p, consistent with a role for this protein in formation of inhibitory TORC1 “body” formation during glucose and nitrogen starvation (71, 72).

Of note, there is no obvious homolog of Tco89p in mammals. However, since Tco89p is intrinsically disordered, due to the absence of structural constraints, it would be expected to have evolved rapidly and to have undergone positive selection at specific sites, resulting in the acquisition of new functions (73), leading us to postulate that the mammalian functional homolog is divergent at the sequence level. Notwithstanding this potential lack of recognizable sequence identity, our yeast OspB phenotype of sensitization to TOR inhibition is similar to our prior finding of OspB-mediated sensitization to rapamycin in fibroblasts (29), bolstering the relevance of the yeast model.

The potential utility of identifying a substrate of a microbial protease in a heterologous system, as we did here for OspB, is exemplified by the work leading to the identification of the physiological ligand of the NLRP1 inflammasome. The Bacillus anthracis lethal factor protease efficiently cleaves a disordered linker in murine NLRP1B and rat NLRP1, releasing an arginine N-degron, degradation of which leads to inflammasome activation in macrophages and pyroptotic cell death (74–76). Anthrax is primarily a pathogen of humans, and lethal factor does not cleave the human NLRP1 homolog (77). Yet, these studies facilitated the recent determination that dependence on functional degradation is a conserved feature of NLRP1 activation (78–80), and the subsequent molecular identification of enteroviral proteases as the physiological activators of the human NLRP1 inflammasome, in which cleavage of the NLRP1 disordered linker generates a glycine N-degron (81, 82). By analogy, through determination of the activity of OspB, our study provides an important insight into its substrate specificity and phenotypic impact, which will facilitate identification of mammalian substrates.

## MATERIALS AND METHODS

### Strains and media.

All strains, plasmids, and primers are listed in Table S3, respectively. E. coli DH10B (83) was used as the routine cloning host and was grown in Luria broth at 37°C with agitation. S. cerevisiae S288C was used as the heterologous expression host and was routinely cultured at 30°C in yeast extract-peptone-dextrose (YPD) broth or in synthetic selective media (MP Biomedicals) lacking histidine, uracil and/or leucine for auxotrophic selection. 1.5% (wt/vol) agar was added for solid media formulations, and where appropriate, medium was supplemented with 50 μg/mL ampicillin (Sigma, A9518), 2% (wt/vol) d-glucose (Fisher Scientific, D16-10), 2% D-(+)-raffinose (Sigma, R7630), 2% (wt/vol) d-galactose (VWR, 200001-176), 300 μg/mL hygromycin (Gibco, 10687010), 200 μg/mL Geneticin (Sigma, A1720). For TORC1 inhibition, solid medium was supplemented with caffeine (Sigma, C0750) or rapamycin (Sigma, 553211) at 6 mM or 5 nM, respectively, unless stated otherwise. For proteasome inhibition, medium was supplemented with 75 μM MG-132 (Selleck Chemicals, S2619) and 0.003% SDS. Yeast strains were transformed using the standard lithium acetate method. Yeast gene complementation analyses were conducted using low-copy centromeric plasmids expressing the gene of interest from its native promoter unless stated otherwise. Overexpression of *TCO89* was achieved by constitutive expression of the construct from a multicopy vector.

10.1128/mbio.01270-22.10 TABLE S3 Bacterial strains, yeast strains, plasmids, and primers used in this study. Download Table S3, PDF file, 0.2 MB. Copyright © 2022 Wood et al. 2022 Wood et al. https://creativecommons.org/licenses/by/4.0/ This content is distributed under the terms of the Creative Commons Attribution 4.0 International license.

### Bioinformatic analyses.

*In silico* modeling of the tertiary structure of OspB was conducted on the Phyre2 server (84), whereas alignment with the crystal structures of RtxA^VC^ (31) and TcdA (26) was achieved using the CEAlign algorithm within PyMol (Schrödinger, LLC). Protein sequences were retrieved from the nonredundant NCBI database and aligned using MUSCLE (85) to select the regions of interest before manual curation.

### Yeast growth assays and protein extraction.

Individual yeast transformants that constitutively express *ospB* or derivatives containing point mutations were grown in synthetic selective liquid media containing 2% d-glucose. To measure the impact of OspB constructs on growth, yeast cells grown in liquid media were washed and serially diluted 4-fold in phosphate-buffered saline, and 5 μL of each dilution was spotted on synthetic selective solid media with additives as appropriate. Assessment of protein production was from liquid cultures. Here, subcultures were inoculated at OD_600_ 2.0 from overnight cultures and grown for 2 h before harvesting for SDS-PAGE analysis using the alkaline lysis method (86). Where construct induction was required, yeast strains were subcultured in 2% raffinose for 2 h, before supplementation with 2% galactose. Samples were harvested after 4 h of protein expression. For proteasome inhibition, yeast strains were subcultured in glucose for 2 h before treatment with MG-132 or DMSO control for 3 h.

### Yeast library screening.

To screen for suppressors of OspB-mediated toxicity in S. cerevisiae by yeast gene overexpression, the strain BY4742 pAG413GPD-*ospB* was mated with the haploid GST-fusion yeast overexpression library (Dharmacon, YSC4423) on YPD. The resulting diploids were selected by plating on noninducing synthetic selective media containing 2% d-glucose. The screen was conducted by spotting in quadruplicate on inducing synthetic selective solid media containing 2% d-galactose (to induce strong expression of yeast genes in the library) and 6 mM caffeine. All steps in the screen were conducted in an automated manner as described previously (18). Suppressors were classified as strains that displayed qualitatively moderate to robust growth of all four spots on the caffeine plate at 4 days after pinning. To screen for S. cerevisiae host factors required for OspB-dependent growth inhibition, we screened the MAT**a** haploid deletion library (Horizon, YSC1053) as previously described (17), but with transformation of the plasmid pAG413GAL-*ospB* and assessment of growth on synthetic selective solid media containing 2% d-galactose (to induce expression of *ospB*) and 6 mM caffeine at 3 days after pinning.

### Cell culture and transfection.

HEK293T (ATCC) and mouse embryonic fibroblast cells (29) were maintained in Dulbecco’s modified Eagle medium (DMEM) (Gibco) supplemented with 10% (vol/vol) fetal bovine serum at 37°C with 5% CO_2_. Cells were transfected with plasmids using FuGENE6 (Promega) according to the manufacturer’s instructions, and experimental samples were analyzed 24 to 48 h after transfection.

### SDS-PAGE and immunoblotting.

For immunoblot analysis, protein samples were separated by SDS-PAGE, transferred to nitrocellulose membranes and detected by Western blot analysis using standard procedures. The antibodies used were peroxidase-conjugated anti-β-actin (Sigma, A3854; diluted to 1:10 000), anti-α-tubulin (Santa Cruz, sc-53030; diluted to 1:1000), anti-FLAG (Sigma, F3165; diluted to 1:1000), anti-myc (EMD Millipore 05-724; diluted to 1:1000), anti-HA (Biolegend, 901501, diluted to 1:1000) and anti-OspB (diluted to 1:10 000). The rabbit anti-OspB antibody was generated (Covance Inc.) against a 14-mer peptide of OspB located 18 residues from the C-terminus.

### Data availability.

All relevant data are included in the manuscript and will be made available by request to the corresponding author, marcia.goldberg@mgh.harvard.edu.
