# Supplementary material for: The Shigella Spp. Type III Effector Protein OspB Is a Cysteine Protease
Source: mBio. 2022 May 31;13(3):e01270-22. doi: 10.1128/mbio.01270-22 (PMC9239218; doi:10.1128/mbio.01270-22)
Supplement: FIG S1 [file mbio.01270-22-sf001.pdf]

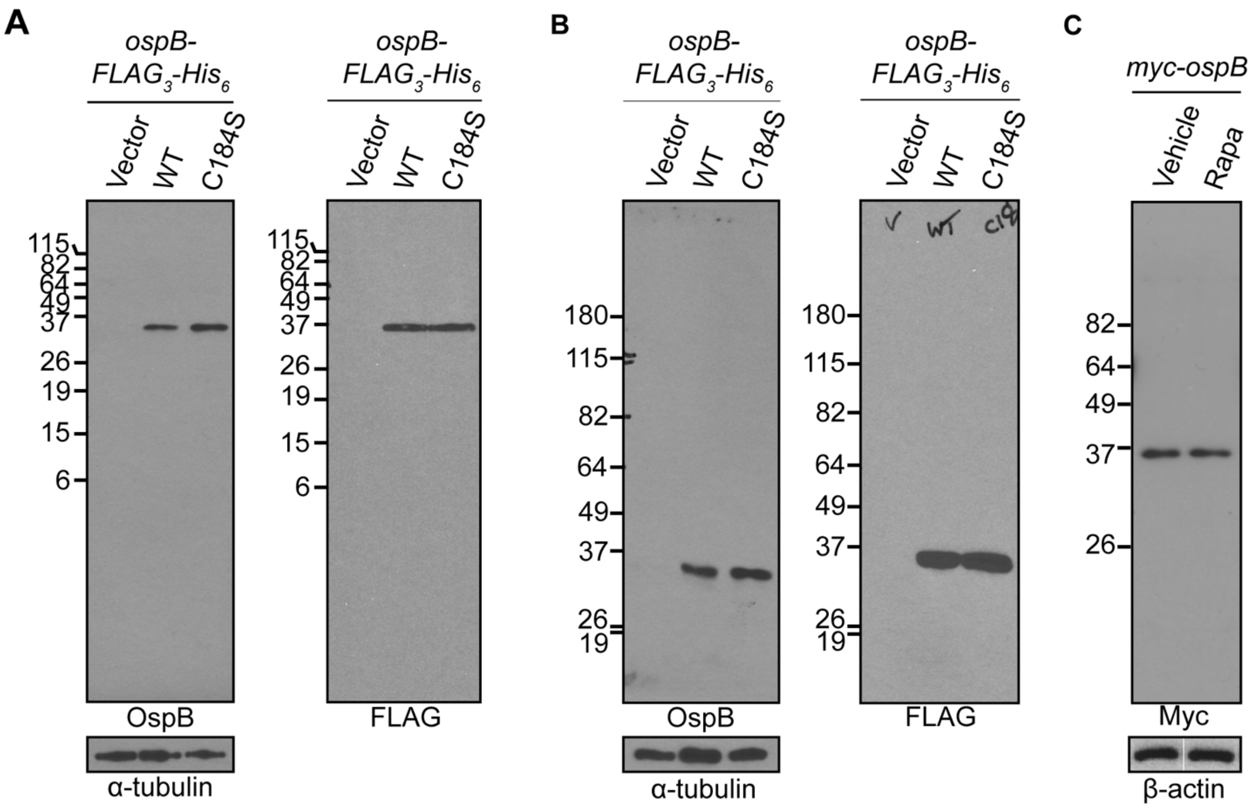

FIG S1 Absence of evidence of processing of OspB in cell lysates. (A) Wild type (WT) OspB and OspB (C184S) expressed in yeast, detected by immunoblotting with anti-OspB and anti-FLAG antibodies after separation on a 15% SDS-PAGE gel. α-tubulin serves as a loading control. (B) Samples from panel (A) separated on a 7.5% SDS-PAGE gel and probed as in panel (A). (C) Transfection of mouse embryonic fibroblasts with pCMV-*myc-ospB*. Cells were treated with rapamycin (10 nM) (rapa) or a DMSO vehicle control. Myc-OspB detected by immunoblotting with an anti-myc antibody after separation on a 10% SDS-PAGE gel. β-actin serves as the loading control; bands from a single blot. MW in kD to the left of blots.
