# Supplementary figures and images for: The Shigella Spp. Type III Effector Protein OspB Is a Cysteine Protease
Source: mBio. 2022 May 31;13(3):e01270-22. doi: 10.1128/mbio.01270-22 (PMC9239218; doi:10.1128/mbio.01270-22)

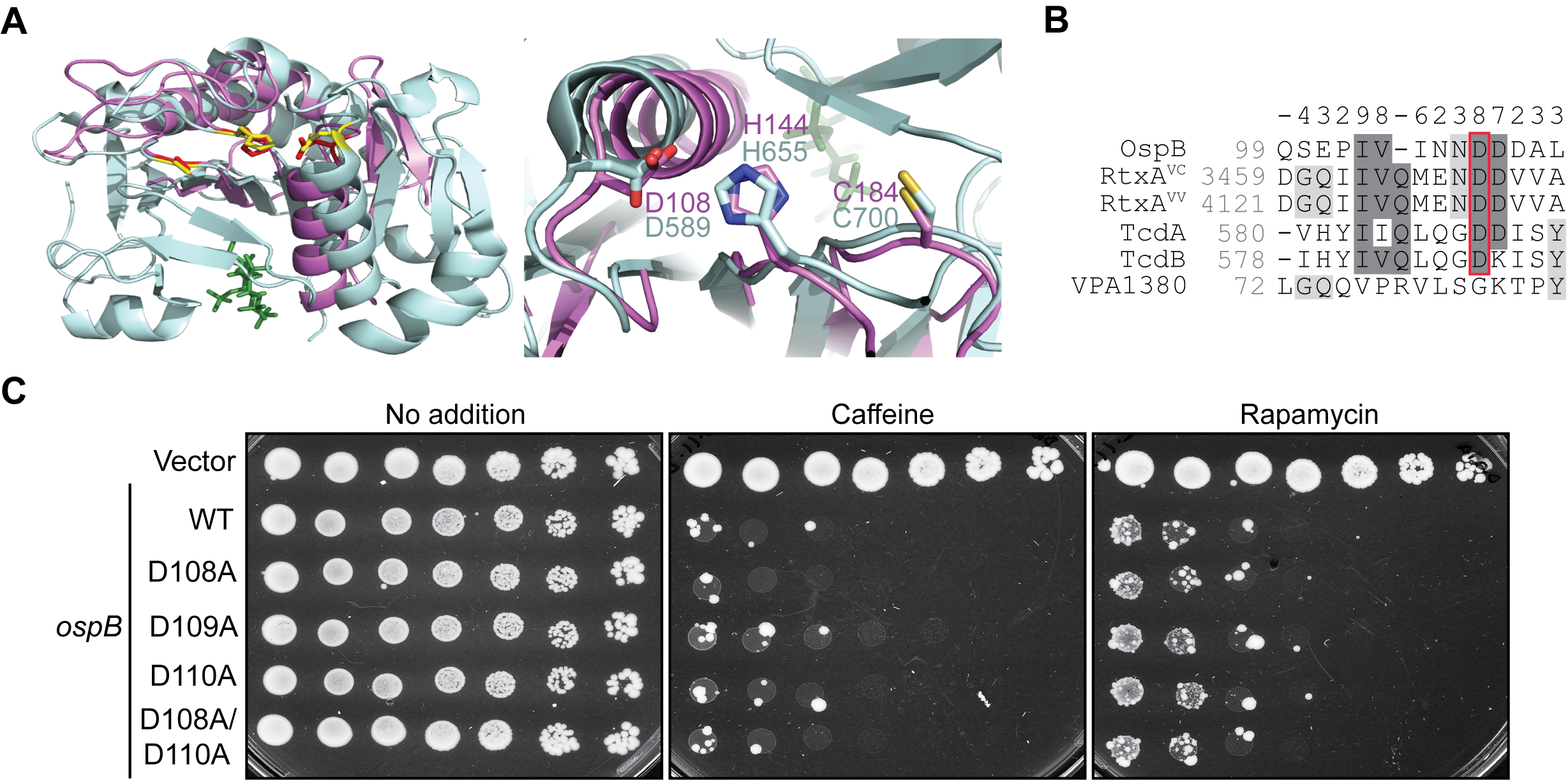

Supplement: FIG S2 [file mbio.01270-22-sf002.tif]
