# Supplementary material for: The Shigella Spp. Type III Effector Protein OspB Is a Cysteine Protease
Source: mBio. 2022 May 31;13(3):e01270-22. doi: 10.1128/mbio.01270-22 (PMC9239218; doi:10.1128/mbio.01270-22)
Supplement: TABLE S1 [file mbio.01270-22-st001.pdf]

TABLE S1 Genes identified as required for OspB-dependent sensitization of yeast to caffeine. Individual yeast strains lacking each of these genes displayed robust growth when expressing *ospB* in the presence of caffeine. Robust growth was defined quantitatively as previously described (1). Genes encoding proteins with a characterized role in TORC1 signaling are shown in bold.

Gene	Locus Tag	Gene	Locus Tag	Gene	Locus Tag	Gene	Locus Tag
-	*YBR085C-A*	*CIT3*	*YPR001W*	*MFA2*	*YNL145W*	*SAK1*	*YER129W*
-	*YDR442W*	*CTF19*	*YPL018W*	*NAT3*	*YPR131C*	***SAP185***	***YJL098W***
-	*YEL025C*	*DCV1*	*YFR012W*	*NGR1*	*YBR212W*	*SAP30*	*YMR263W*
-	*YGL041C-B*	*DOA1*	*YKL213C*	***NPR1***	***YNL183C***	*SIF2*	*YBR103W*
-	*YGR025W*	*DSK2*	*YMR276W*	*NTA1*	*YJR062C*	*SKI8*	*YGL213C*
-	*YLR434C*	*ECM23*	*YPL021W*	*PAF1*	*YBR279W*	*SNA3*	*YJL151C*
-	*YMR052C-A*	*ETR1*	*YBR026C*	*PBI2*	*YNL015W*	*SOD1*	*YJR104C*
-	*YNL040W*	*FOX2*	*YKR009C*	*PDB1*	*YBR221C*	*SOL1*	*YNR034W*
-	*YNL057W*	***FPR1***	***YNL135C***	*PEP12*	*YOR036W*	*SPO7*	*YAL009W*
-	*YNL140C*	***GLN3***	***YER040W***	*PEP7*	*YDR323C*	***STP1***	***YDR463W***
-	*YNL195C*	*HAT1*	*YPL001W*	*PET191*	*YJR034W*	*SWA2*	*YDR320C*
-	*YNL296W*	*HOS2*	*YGL194C*	*PUB1*	*YNL016W*	*SYF2*	*YGR129W*
-	*YNR005C*	*HSE1*	*YHL002W*	*RAD6*	*YGL058W*	***TIP41***	***YPR040W***
AIM22	*YJL046W*	*HTD2*	*YHR067W*	*RBS1*	*YDL189W*	*UBA3*	*YPR066W*
ALF1	*YNL148C*	*IPK1*	*YDR315C*	*RIB1*	*YBL033C*	*UBP3*	*YER151C*
APE2	*YKL157W*	*IRC21*	*YMR073C*	*RNR1*	*YER070W*	*UBR1*	*YGR184C*
ATE1	*YGL017W*	*IWR1*	*YDL115C*	*RPN4*	*YDL020C*	*VIP1*	*YLR410W*
BRE5	*YNR051C*	*KCC4*	*YCL024W*	*RPS12*	*YOR369C*	*VPS13*	*YLL040C*
BRR1	*YPR057W*	*LIP2*	*YLR239C*	***RRD1***	***YIL153W***		
BUL1	***YMR275C***	*LPD1*	*YFL018C*	***RTG3***	***YBL103C***		
CBT1	*YKL208W*	*MCT1*	*YOR221C*	*SAC3*	*YDR159W*		

Reference

1. Kramer RW, Slagowski NL, Eze NA, Giddings KS, Morrison MF, Siggers KA, Starnbach MN, Lesser CF. 2007. Yeast functional genomic screens lead to

identification of a role for a bacterial effector in innate immunity regulation. PLoS Pathog 3:e21.
