# Supplementary material for: The Shigella Spp. Type III Effector Protein OspB Is a Cysteine Protease
Source: mBio. 2022 May 31;13(3):e01270-22. doi: 10.1128/mbio.01270-22 (PMC9239218; doi:10.1128/mbio.01270-22)
Supplement: FIG S3 [file mbio.01270-22-sf003.pdf]

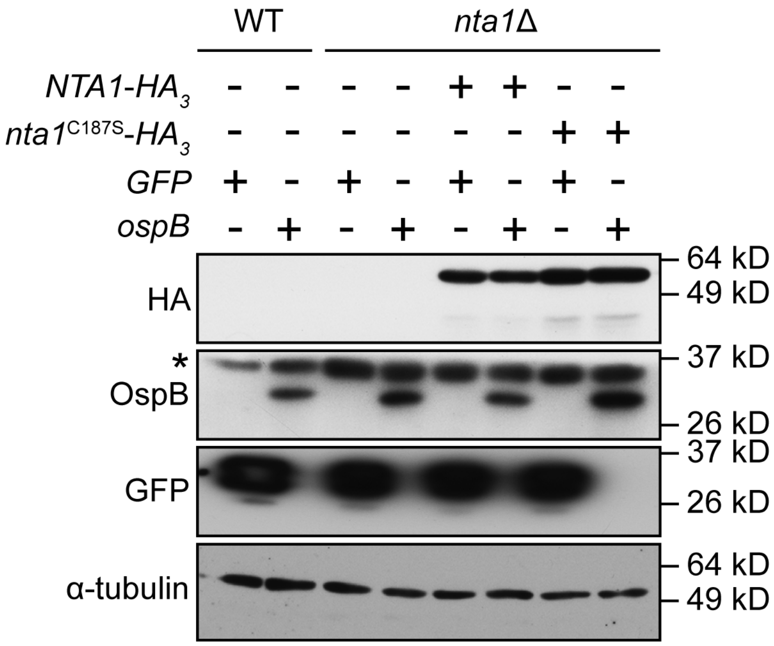

FIG S3 Production of Nta1p variants. Abundance of the WT and catalytically inactive Nta1p variant in cells that express OspB or GFP control. Western blot. Asterisk, a non-specific protein recognized by the anti-OspB antibody. α-tubulin, loading control (*n* = 3).
