# Supplementary material for: The Shigella Spp. Type III Effector Protein OspB Is a Cysteine Protease
Source: mBio. 2022 May 31;13(3):e01270-22. doi: 10.1128/mbio.01270-22 (PMC9239218; doi:10.1128/mbio.01270-22)
Supplement: FIG S4 [file mbio.01270-22-sf004.pdf]

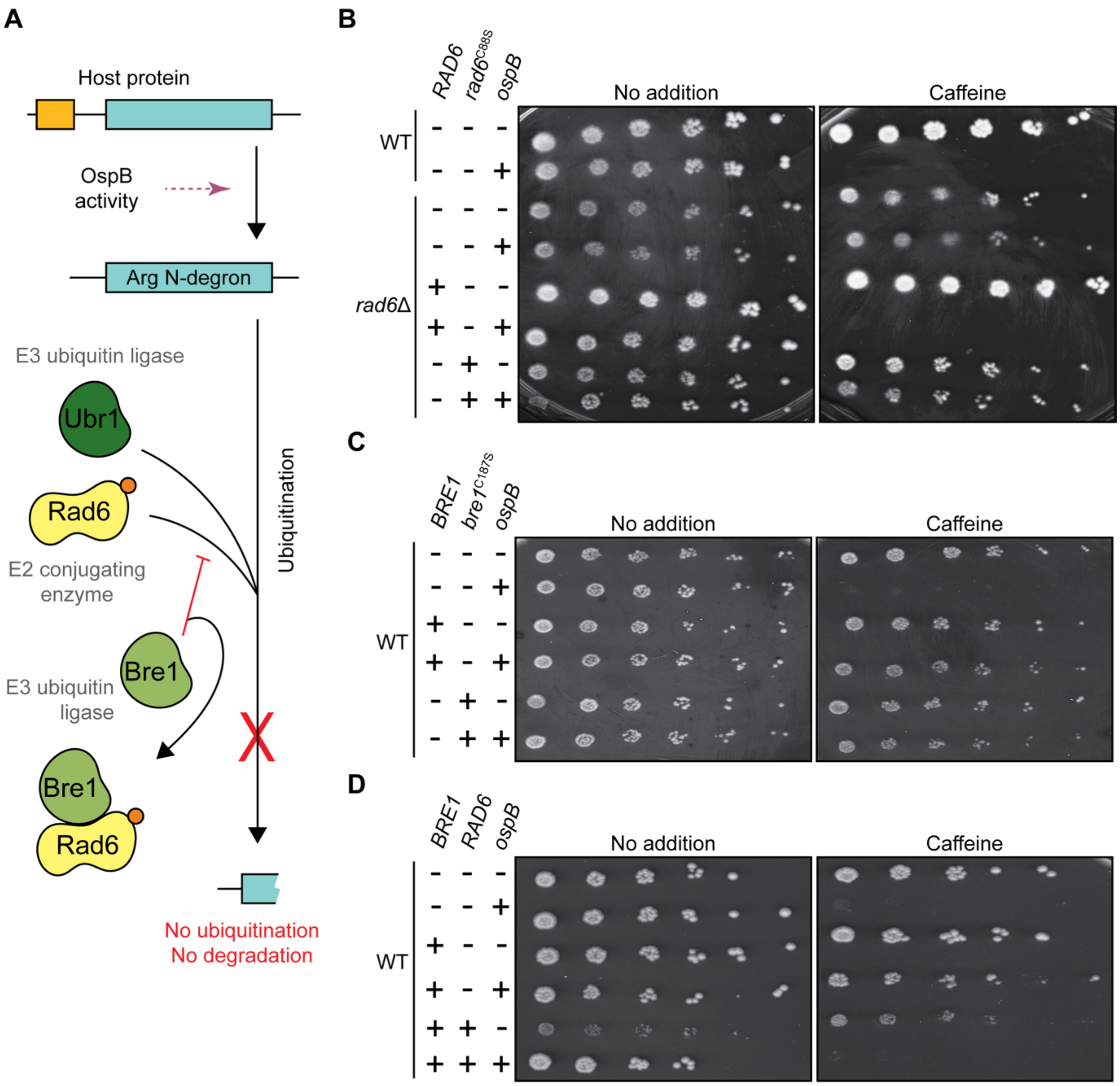

FIG S4 Bre1p rescues growth inhibition through sequestration of arginine N-degron pathway component Rad6p. (A) Schematic of the proposed mechanism of Bre1-mediated suppression of OspB-dependent growth inhibition. (B) Growth of wild type (WT) or *rad6* deletion yeast strains expressing *ospB* or vector control. Serial dilutions spotted on media with or without caffeine (*n* = 3). (C) Growth of yeast strains expressing *ospB* or vector control in the presence or absence of indicated multi-copy *BRE1* alleles. Serial dilutions spotted on media with or without caffeine (*n* = 3). (D) Growth of WT yeast expressing *ospB* or vector control in the presence or absence of additional gene copies of *BRE1* and/or *RAD6*. Serial dilutions spotted on media with or without caffeine (*n* = 3).
