# Supplementary material for: The Shigella Spp. Type III Effector Protein OspB Is a Cysteine Protease
Source: mBio. 2022 May 31;13(3):e01270-22. doi: 10.1128/mbio.01270-22 (PMC9239218; doi:10.1128/mbio.01270-22)
Supplement: TABLE S2 [file mbio.01270-22-st002.pdf]

TABLE S2 Genes identified as suppressors of OspB-dependent sensitization of yeast to caffeine. Expression of these genes from a multi-copy vector under an inducible promoter rescued the growth of wild type yeast co-expressing *ospB* when grown in the presence of caffeine. Assessment of growth was conducted in a qualitative manner.

Gene	Locus Tag	Gene	Locus Tag	Gene	Locus Tag
-	*YAR023C*	*FMT1*	*YBL013W*	*QCR6*	*YFR033C*
-	*YBL100C*	*GAL11*	*YOL051W*	*RIM11*	*YMR139W*
-	*YBR116C*	*HIS3*	*YOR202W*	*SEC3*	*YER008C*
-	*YBR284W*	*HSP60*	*YLR259C*	*SIW14*	*YNL032W*
BRE1	*YDL074C*	*JJJ3*	*YJR097W*	*SLX8*	*YER116C*
CLN3	*YAL040C*	*LAT1*	*YNL071W*	*TCB1*	*YOR086C*
COS3	*YML132W*	*LSB1*	*YGR136W*	*TKL2*	*YBR117C*
CSM1	*YCR086W*	*MTC6*	*YHR151C*	*UBC1*	*YDR177W*
CYC3	*YAL039C*	*PBY1*	*YBR094W*	*UMP1*	*YBR173C*
DDI1	*YER143W*	*PPE1*	*YHR075C*	*YUH1*	*YJR099W*
DDP1	*YOR163W*	*PRX1*	*YBL064C*		
EMA17	*YIL029C*	*PTC3*	*YBL056W*		
