# Supplementary material for: The Shigella Spp. Type III Effector Protein OspB Is a Cysteine Protease
Source: mBio. 2022 May 31;13(3):e01270-22. doi: 10.1128/mbio.01270-22 (PMC9239218; doi:10.1128/mbio.01270-22)
Supplement: FIG S5 [file mbio.01270-22-sf005.pdf]

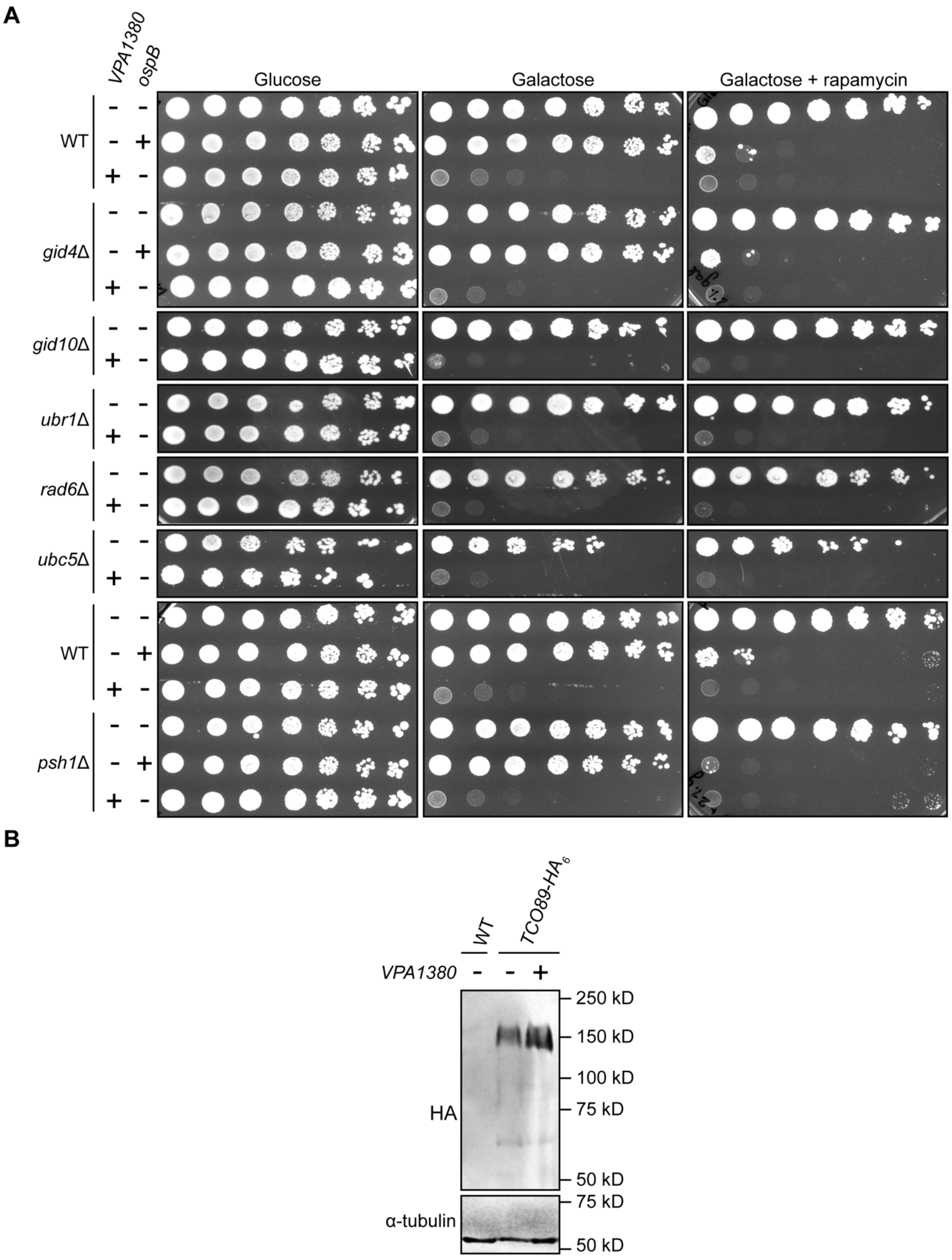
A
VPA1380
ospB
Glucose
Galactose
Galactose + rapamycin
WT
gid4Δ
gid10Δ
ubr1Δ
rad6Δ
ubc5Δ
WT
psh1Δ
B
WT
TCO89-HA6
VPA1380
250 kD
150 kD
100 kD
75 kD
50 kD
HA
α-tubulin
75 kD
50 kD

FIG S5 N-degron pathways are not required for VPA1380 toxicity phenotype, and VPA1380 does not cleave Tco89p. (A) The effect on growth of *ospB* and *VPA1380* expression in wild type (WT) yeast, proline N-degron pathway mutants (lacking N-recognins Gid4 or Gid10), arginine N-degron pathway mutants (lacking amidase Nta1, E3 ubiquitin ligase Ubr1, or E2 conjugating enzymes Ubc5 or Rad6), or a formyl-methionine N-degron pathway mutant (lacking E3 ubiquitin ligase Psh1). Serial dilutions spotted on media in conditions that repress (glucose) or induce (galactose) construct expression. Supplementation of media with rapamycin where indicated (n = 2). (B) Absence of cleavage of Tco89p by VPA1380. Western blot. α-tubulin is the loading control (n = 2).
