# Supplementary material for: The Shigella Spp. Type III Effector Protein OspB Is a Cysteine Protease
Source: mBio. 2022 May 31;13(3):e01270-22. doi: 10.1128/mbio.01270-22 (PMC9239218; doi:10.1128/mbio.01270-22)
Supplement: FIG S6 [file mbio.01270-22-sf006.pdf]

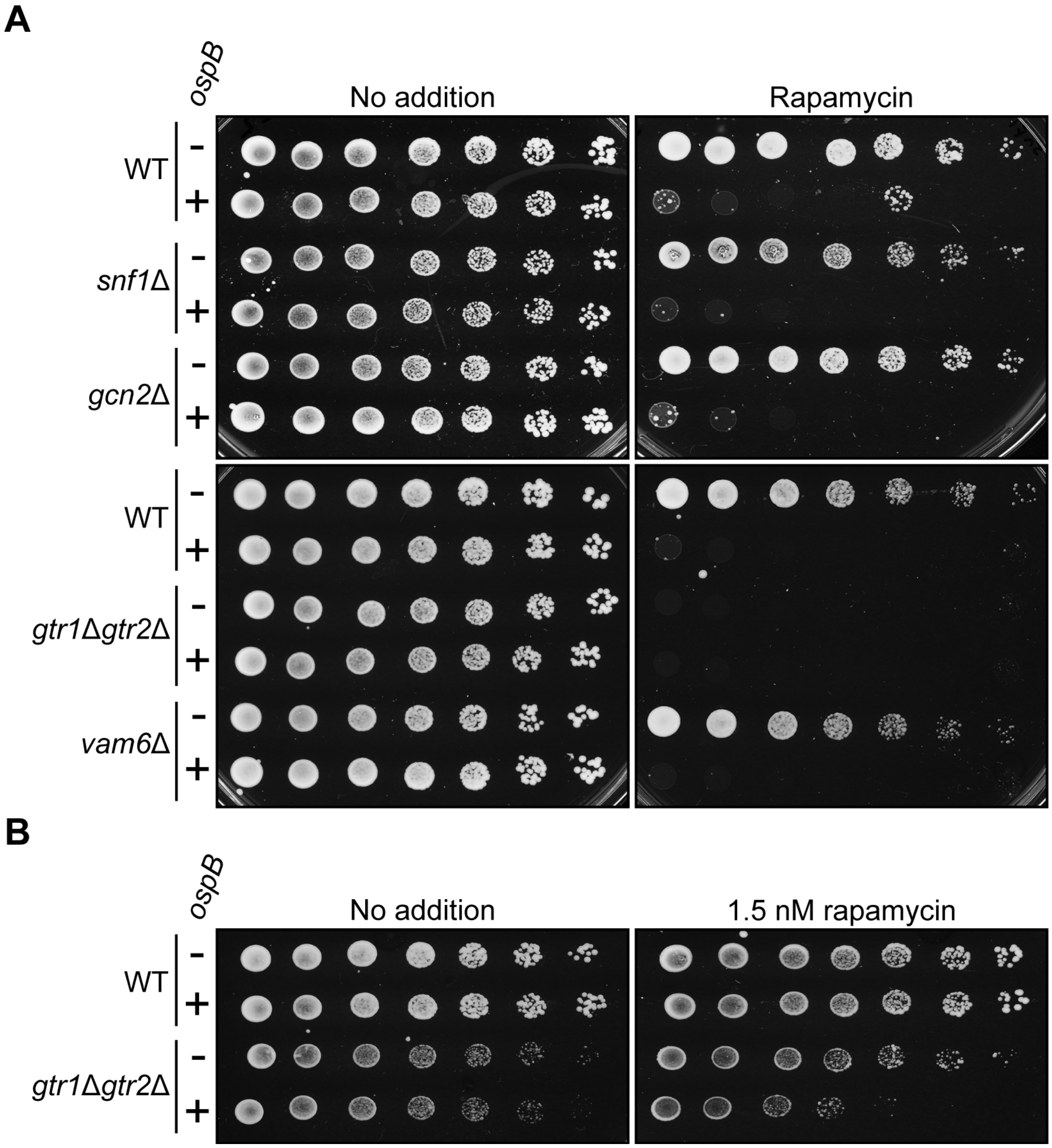

FIG S6 OspB does not act upstream of TORC1. (A) Effect of *ospB* expression on the growth of yeast strains lacking genes involved in nutrient sensing upstream of TORC1 signaling compared with wild type (WT) yeast. Serial dilutions spotted on media with or without rapamycin (n = 3). (B) Growth of WT and a *gtr*1Δ*gtr*2Δ yeast strain expressing *ospB*, plated on solid media containing no additive or 1.5 nM rapamycin. This reduced rapamycin concentration was used due to sensitivity of the *gtr*1Δ*gtr*2Δ mutant to it (n = 3).
