# Supplementary material for: The Shigella Spp. Type III Effector Protein OspB Is a Cysteine Protease
Source: mBio. 2022 May 31;13(3):e01270-22. doi: 10.1128/mbio.01270-22 (PMC9239218; doi:10.1128/mbio.01270-22)
Supplement: FIG S7 [file mbio.01270-22-sf007.pdf]

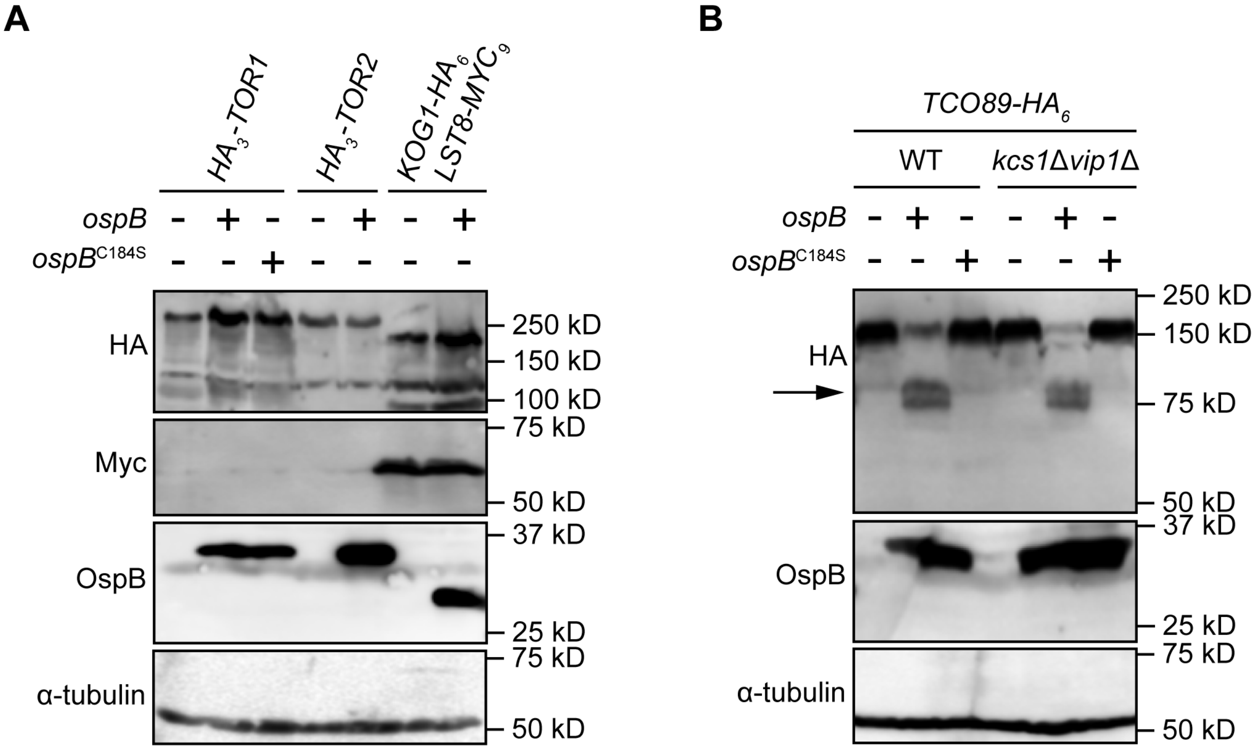

FIG S7 Independence of OspB protease activity from components of TORC1 other than Tco89p and inositol pyrophosphates. (A) Lack of cleavage of components of the yeast TORC1 complex by OspB. TORC1 proteins (Tor1p, Tor2p, Kog1p and Lst8), tagged and at their native loci. The OspB construct produced by the *KOG1-HA$_6$ LST8-myc$_9$* strain is untagged, whereas in all other strains, OspB has a C-terminal FLAG$_3$-His$_6$ tag. Western blot. α-tubulin, loading control (*n* =3). (B) Cleavage of Tco89p in wild type and *kcs*1Δ*vip1*Δ yeast, in the presence of OspB, OspB(C184S) or vector control. Western blot. α-tubulin, loading control. Arrow, the Tco89p C-terminal cleavage product (*n* = 3).
