# Supplementary material for: The Shigella Spp. Type III Effector Protein OspB Is a Cysteine Protease
Source: mBio. 2022 May 31;13(3):e01270-22. doi: 10.1128/mbio.01270-22 (PMC9239218; doi:10.1128/mbio.01270-22)
Supplement: TABLE S3 [file mbio.01270-22-st003.pdf]

TABLE S3 Bacterial strains, yeast strains, plasmids, and primers used in this study.

Strain	Description	Source
E. coli		
DH10B	str. K-12 F^{-} Δ(*ara-leu*)*7697*[Δ(*rapA'-cra'*)] Δ(*lac*)*X74*[Δ(*'yahH-mhpE*)] duplication(514341-627601)[*nmpC-gltI*] *galK16 galE15* e14^{-}(*icd*^{WT} *mcrA*) φ80d*lacZΔM15 recA1 relA1 endA1* Tn*10.10 nupG rpsL150*(Str^{R}) *rph*$^{+}$ *spoT 1 Δ(mrr-hsdRMS-mcrBC)* λ^{-} Missense(*dnaA glmS glyQ lpxK mreC murA*) Nonsense(*chiA gatZ fhuA*? *yigA ygcG*) Frameshift(*flhC mglA fruB*)	Laboratory collection
S. cerevisiae		
BY4741	MATa *his3Δ1 leu2Δ0 met15Δ0 ura3Δ0*	Laboratory collection
ipk1Δ	MATa *his3Δ1 leu2Δ0 met15Δ0 ura3Δ0 ipk1::LEU2*	This study
kcs1Δvip1Δ	MATa *his3Δ1 leu2Δ0 met15Δ0 ura3Δ0 kcs1::kanMX4 vip1::LEU2*	This study
TCO89-HA_6	*MATa his3Δ1 leu2Δ0 met15Δ0 ura3Δ0 TCO89-6xHA::hphNT1*	This study
ipk1Δ TCO89-HA_6	MATa *his3Δ1 leu2Δ0 met15Δ0 ura3Δ0 ipk1::LEU2 TCO89-6xHA::hphNT1*	This study
nta1ΔTCO89-HA_6	MATa *his3Δ1 leu2Δ0 met15Δ0 ura3Δ0 nta1::kanMX4 TCO89-6xHA::hphNT1*	This study
gtr1Δgtr2Δ	MATa *his3Δ1 leu2Δ0 met15Δ0 ura3Δ0 gtr1::kanMX4 gtr2::LEU2*	This study
nta1Δ	MATa *his3Δ1 leu2Δ0 met15Δ0 ura3Δ0 nta1::kanMX4*	Horizon MATa yeast knock-out collection
ate1Δ	MATa *his3Δ1 leu2Δ0 met15Δ0 ura3Δ0 ate1::kanMX4*	Horizon MATa yeast knock-out collection
ubr1Δ	MATa *his3Δ1 leu2Δ0 met15Δ0 ura3Δ0 ubr1::kanMX4*	Horizon MATa yeast knock-out collection
rad6Δ	MATa *his3Δ1 leu2Δ0 met15Δ0 ura3Δ0 rad6::kanMX4*	Horizon MATa yeast knock-out collection
pib2Δ	MATa *his3Δ1 leu2Δ0 met15Δ0 ura3Δ0 pib2::kanMX4*	Horizon MATa yeast knock-out collection
tco89Δ	MATa *his3Δ1 leu2Δ0 met15Δ0 ura3Δ0 tco89::kanMX4*	Horizon MATa yeast knock-out collection
gid4Δ	MATa *his3Δ1 leu2Δ0 met15Δ0 ura3Δ0 gid4::kanMX4*	Horizon MATa yeast knock-out collection
gid10Δ	MATa *his3Δ1 leu2Δ0 met15Δ0 ura3Δ0 gid10::kanMX4*	Horizon MATa yeast knock-out collection
psh1Δ	MATa *his3Δ1 leu2Δ0 met15Δ0 ura3Δ0 psh1::kanMX4*	Horizon MATa yeast knock-out collection
ubc5Δ	MATa *his3Δ1 leu2Δ0 met15Δ0 ura3Δ0 ubc5::kanMX4*	Horizon MATa yeast knock-out collection
snf1Δ	MATa *his3Δ1 leu2Δ0 met15Δ0 ura3Δ0 snf1::kanMX4*	Horizon MATa yeast knock-out collection
gcn2Δ	MATa *his3Δ1 leu2Δ0 met15Δ0 ura3Δ0 gcn2::kanMX4*	Horizon MATa yeast knock-out collection
vam6Δ	MATa *his3Δ1 leu2Δ0 met15Δ0 ura3Δ0 vam6::kanMX4*	Horizon MATa yeast knock-out collection

KOG1-HA$_6$ LST8-MYC$_9$	MATa *his3Δ1 leu2Δ0 met15Δ0 ura3Δ0 ipk1::LEU2 KOG1-6xHA::hphNT1 LST8-9xMYC::natNT2*	(1)
W303a *HA$_3$-TOR1*	MATa *leu2-3112 ura3-52 can1- 100 ade2-1 his3-11 trp1-11 HISMX6::3xHA-TOR1*	(2)
W303a *HA$_3$-TOR2*	MATa *leu2-3112 ura3-52 can1- 100 ade2-1 his3-11 trp1-11 HISMX6::3xHA-TOR2*	(2)
**Plasmid**	**Purpose**	**Source**
pAG413GAL-*ospB*	Yeast plasmid conditionally producing OspB	(3)
pAG413GPD	Yeast centromeric parental vector	Susan Lindqvist
pAG413GPD-*EGFP*	Yeast plasmid producing EGFP	Susan Lindqvist
pAG413GPD-*ospB*	Yeast plasmid producing OspB	This study
pAG413GPD-*ospB*(C184S)	Yeast plasmid producing OspB(C184S)	This study
pAG413GPD-*ospB*(D108A)	Yeast plasmid producing OspB(D108A)	This study
pAG413GPD-*ospB*(D108A/D110A)	Yeast plasmid producing OspB(D108A/D110A)	This study
pAG413GPD-*ospB*(D109A)	Yeast plasmid producing OspB(D109A)	This study
pAG413GPD-*ospB*(D110A)	Yeast plasmid producing OspB(D110A)	This study
pAG413GPD-*ospB*(H144A)	Yeast plasmid producing OspB(H144A)	This study
pAG415	Yeast centromeric parental vector	Susan Lindqvist
pAG415GAL-*ospB*	Yeast plasmid conditionally producing OspB	This study
pAG415-*NTA1*	Yeast plasmid expressing *NTA1* with native promoter and terminator	This study
pAG415-*nta1*(C187S)	Yeast plasmid expressing *nta1*(C184S) with native promoter and terminator	This study
pAG415-*nta1*(C187S)-*HA$_3$*	Yeast plasmid expressing *nta1*(C184S) from native promoter	This study
pAG415-*NTA1-HA$_3$*	Yeast plasmid expressing *NTA1* from native promoter	This study
pAG415-*RAD6*	Yeast plasmid expressing *RAD6* with native promoter and terminator	This study
pAG415-*rad6*(C88S)	Yeast plasmid expressing *rad6*(C88S) with native promoter and terminator	This study
pAG415-*TCO89-HA$_3$*	Yeast plasmid expressing *TCO89* from native promoter	This study
pAG415GAL-*VPA1380*	Yeast plasmid conditionally producing VPA1380	This study
pCMV-*FLAG*	Mammalian expression vector	Laboratory collection
pCMV-*FLAG$_3$-TCO89*	Mammalian expression vector expressing *TCO89*	This study
pCMV-*myc*	Mammalian expression vector	Laboratory collection
pCMV-*myc-ospB*	Mammalian expression vector producing OspB	This study

pCMV-*myc-ospB*(C184S)	Mammalian expression vector producing OspB(C184S)	This study
pRS313	Yeast centromeric parental vector	(4)
pRS313-*IPK1*	Yeast plasmid expressing *IPK1* with native promoter and terminator	This study
pRS316	Yeast centromeric parental vector	(4)
pRS316GAL-*GST-His*$_6$	Yeast plasmid for conditional expression	This study
pRS316GAL-*GST-His*$_6$*-BRE1*	Yeast plasmid for conditional expression of *BRE1*	This study
pRS316GAL-*GST-His*$_6$*-bre1*(C663S)	Yeast plasmid for conditional expression of *bre1*(C663S)	This study
pRS316GAL-*ospB-FLAG*$_3$*-His*$_6$	Yeast plasmid conditionally producing OspB	This study
pRS316GAL-*VPA1380-FLAG*$_3$*-His*$_6$	Yeast plasmid conditionally producing VPA1380	This study
pRS425	Yeast 2-micron vector	(5)
pRS425GPD-*RFP-TCO89-FLAG*$_3$	Yeast multicopy vector overexpressing *TCO89*	This study
pYM16	Yeast vector providing template for chromosomal insertion of *6xHA* tag	Euroscarf

**Primer**	**Sequence**	**Purpose**
HE107	ACCAGTCGAAAATTGTCAGAGATAAGTTCCTTTTTTGAAAAGAAAGATCGTAACTGTGGGAATACTCAGG	Forward primer for amplification of *LEU2* marker from pRS315 to insert into *IPK1* locus
HE108	TAATGTATGTGCATCTGCCAGTACCAAAGGTGGAAAGAAAAGTATACAGTTTTAAGCAAGGATTTTC	Reverse primer for amplification of *LEU2* marker from pRS315 to insert into IPK1 locus
IPK1_F	CACGTAGGAAAGCGA	Forward screening primer for generation of *ipk1* mutant
IPK1_R	CCCTTCGTTGAATATCG	Reverse screening primer for generation of *ipk1* mutant
HE088	CATACAAATTCAAAAGCATCTCGTAGCATATTAATATATTGCAGAAGGTCGAGCAGACAAGCCCGTCAG	Forward primer for amplification of *LEU2* marker from pRS315 to insert into *VIP1* locus
HE090	TAAATACTTATTTAGTTTTGGGTTACTAAATTAAAAATTGGGTGTGATCACTCCTTACGCATCTGTG	Reverse primer for amplification of *LEU2* marker from pRS315 to insert into *VIP1* locus
VIP1_F	TAGCAATCTCATCGCG	Forward screening primer for generation of *vip1* mutant
VIP1_R	CGAAAACTCCGGACCTAAA	Reverse screening primer for generation of *vip1* mutant
TW187	ATGGAAAATGTGGGCTACATGCATACACAGCCACAACAAAGTCGTACGCTGCAGGTC	Forward primer for tagging endogenous *TCO89* gene at 3' -end

TW188	ATTAGCTACTCTTTTAAACTGTGTGCTTCGTGTTGGTTGTTTGGAGACCGGCAGATCC	Reverse primer for tagging endogenous *TCO89* gene at 3' -end
TW217	TAACAAAACTCCAGGACAACGGTACTAATACACATACAACTAACTGTGGGAATACTCAGG	Forward primer for amplification of *LEU2* marker from pRS315 to insert into *GTR2* locus
TW218	TCTATATACCCTAATATTTTCATGCCTTACGTCTTCTTTTTTAAGCAAGGATTTTCTTAA	Reverse primer for amplification of *LEU2* marker from pRS315 to insert into *GTR2* locus
TW219	AACCGATTAACATCCACAGA	Forward screening primer for generation of *gtr2* mutant
TW220	AAAACTTTGGCACCTCTGTA	Reverse screening primer for generation of *gtr2* mutant
HE008	GTAAAACGACGGCCAGT	Forward screening primer for yeast pRS suite vectors (M13F)
HE009	GGAAACAGCTATGACCATG	Reverse screening primer for yeast pRS suite vectors (M13R-like)
HE001	CCGTTTTACTTCAAGCGGCTCCGCTGATAAAGTGG	Forward primer for amplification of *ospB* (C184S) mutation by Quikchange
HE002	CCACTTTATCAGCGGAGCCGCTTGAAGTAAAACGG	Reverse primer for amplification of *ospB* (C184S) mutation by Quikchange
HE003	GGTTTATATTCTTGGGGCCGGTAGTCCTGGTTCTCATC	Forward primer for amplification of *ospB* (H144A) mutation by Quikchange
HE004	GATGAGAACCAGGACTACCGGCCCCAAGAATATAAACC	Reverse primer for amplification of *ospB* (H144A) mutation by Quikchange
TW017	TAGTAATAAATAATGCTGATGACGCAT	Forward primer for amplification of *ospB* (D108A) mutation by SOEing PCR
TW018	ATGCGTCATCAGCATTATTTATTACTA	Reverse primer for amplification of *ospB* (D108A) mutation by SOEing PCR
TW311	TAAATAATGATGCTGACGCATTGAA	Forward primer for amplification of *ospB* (D109A) mutation by SOEing PCR
TW312	TTCAATGCGTCAGCATCATTATTTA	Reverse primer for amplification of *ospB* (D109A) mutation by SOEing PCR
TW313	TAAATAATGATGATGCCGCATTGAA	Forward primer for amplification of *ospB* (D110A) mutation by SOEing PCR
TW314	TTCAATGCGGCATCATCATTATTTA	Reverse primer for amplification of *ospB* (D110A) mutation by SOEing PCR
TW315	TAAATAATGCTGATGCCGCATTGAAT	Forward primer for amplification of *ospB* (D108A/D110A) double mutation by SOEing PCR

TW316	ATTCAATGCGGCATCAGCATTATTTA	Reverse primer for amplification of *ospB* (D108A/D110A) double mutation by SOEing PCR
TW001	TAGCGCCGTCTTTCAGTTCG	Forward screening primer for *nta1* mutant
HE104	AAACATCTACAACATTGCTTCACAA	Reverse screening primer for *nta1* mutant
TW002	CACCAGCTTCGTCGTCCATC	Forward screening primer for *ate1* mutant
TW003	TCGTTTTCACCCCGCGTATT	Reverse screening primer for *ate1* mutant
TW004	ATCATCGTCGTCTCCATCGC	Forward screening primer for *ubr1* mutant
TW005	CCCAGGCGCTACTAAGACCA	Reverse screening primer for *ubr1* mutant
TW006	GCACACGTCGCTAGAACCAA	Forward screening primer for *rad6* mutant
TW007	TGCCCGCACAGAAGAGTACT	Reverse screening primer for *rad6* mutant
TW087	ATGCTGCAGGAATTCGAGCTCTAGCGCCGTCTTTCAGT	Forward primer to amplify *NTA1* with native promoter
TW088	TCACTCGAGTCAGGATCCCCTAAACACTTCAAATTGGACC	Reverse primer to amplify *NTA1* without stop codon for tagging
TW089	AAGTCCATAGAAATACCTATTGATG	Forward primer for amplification of *nta1* (C187S) mutation by SOEing PCR
TW090	GCATCAATAGGTATTTCTATGGAC	Reverse primer for amplification of *nta1* (C187S) mutation by SOEing PCR
TW208	TCACTCGAGTCAGGATCCCGTTCCCTTATCCTTCGG	Reverse primer for amplification of *NTA1* with native terminator and stop codon
MY009	ATGGAGCTCGGATATGGTACCGATGTTGT	Forward primer to amplify *RAD6* with native promoter
MY010	TCACTCGAGAAGCTTCTATCATGATCAGTCTGCTTCGTCGT	Reverse primer for amplification of *RAD6* with stop codon
MY001	GCAAATGGTGAAATTTCTTTGGATAT	Forward primer for amplification of *rad6* (C88S) mutation by SOEing PCR
MY002	TGCAAAATATCCAAAGAAATTTCACC	Reverse primer for amplification of *rad6* (C88S) mutation by SOEing PCR
MY007	ATGATCGATAGATATGACGGCCGAGC	Forward primer for amplification of *BRE1*
MY008	TCAGGGCCCTTACAAGTGCACTGTCAATAAATC	Reverse primer for amplification of *BRE1* with stop codon
MY003	CTATTAAAACCTCTGGCCATGT	Forward primer for amplification of *bre1* (C663S) mutation by SOEing PCR
MY004	AGACATGGCCAGAGGTTT	Reverse primer for amplification of *bre1* (C663S) mutation by SOEing PCR
TW113	ATGGAGCTCGGCTGGCACGATGATTAA	Forward primer to amplify *TCO89* with native promoter

TW114	TCAGCGGCCGCCACTAGTCCTTTGTTGTGGCTGTGT	Reverse primer to amplify *TCO89* without stop codon for tagging
HE041	TGTAGTCGATGTCATGATCCTTGTAATCACCGTCATGGTCCTTGTAGTCGCTAGCATCCAGTTCTTTATTAATAA	Reverse primer 1 for constructing *ospB-3xFLAG-6xHis*
HE042	TAGAGCGATAAGCTTTCAACCATGGTGATGGTGATGATGCTTGTCATCGTCATCCTTGTAGTCGATGTCATGATC	Reverse primer 2 for constructing *ospB-3xFLAG-6xHis*
TW222	ATGTGTACAAGGATATCAGGCCTGTTCATCGAGGAAGGACTTT	Forward primer to amplify *TCO89* without start codon
TW223	TCAGTTAACGCGGCCGCGCTAGCTCTAGATCACCTTTGTTGTGGCTG	Reverse primer to amplify *TCO89* with stop codon for tagging
TW189	ATGGAGCTCCCCCACCTCACAAATCTATT	Forward primer for amplification of *IPK1* with native promoter
TW190	TCACTCGAGTCCCTTACATCCCAATCTTTG	Reverse primer for amplification of *IPK1* with stop codon and native terminator
TW232	ATGTCTAGAGTCGACTCCGGTTCTGCTGCTAGTGGTATGGCCTCCTCCGAG	Forward primer for amplification of *RFP* for *TCO89* overexpression
TW233	TCAGGTACCATCGATAGATCTTCACTACTTGTCATCGTCATC	Reverse primer for amplification of 3x*FLAG* tag for *TCO89* overexpression
KW003	TCAGCTAGCACTAGTATCTAAATCAGATTCTAAGGTAAC	Reverse primer for amplification of *VPA1380* without stop codon

References

1. Chen H, Miller PW, Johnson DL, Laribee RN. 2020. The Ccr4-Not complex regulates TORC1 signaling and mitochondrial metabolism by promoting vacuole V-ATPase activity. PLoS Genet 16:e1009046.
2. Wedaman KP, Reinke A, Anderson S, Yates J, 3rd, McCaffery JM, Powers T. 2003. Tor kinases are in distinct membrane-associated protein complexes in Saccharomyces cerevisiae. Mol Biol Cell 14:1204-20.
3. Slagowski NL, Kramer RW, Morrison MF, LaBaer J, Lesser CF. 2008. A functional genomic yeast screen to identify pathogenic bacterial proteins. PLoS Pathog 4:e9.
4. Sikorski RS, Hieter P. 1989. A system of shuttle vectors and yeast host strains designed for efficient manipulation of DNA in Saccharomyces cerevisiae. Genetics 122:19-27.
5. Christianson TW, Sikorski RS, Dante M, Shero JH, Hieter P. 1992. Multifunctional yeast high-copy-number shuttle vectors. Gene 110:119-22.
